# Biophysical characterization of calcium-binding and modulatory-domain dynamics in a pentameric ligand-gated ion channel

**DOI:** 10.1073/pnas.2210669119

**Published:** 2022-12-08

**Authors:** Marie Lycksell, Urška Rovšnik, Anton Hanke, Anne Martel, Rebecca J. Howard, Erik Lindahl

**Affiliations:** ^a^Department of Biochemistry and Biophysics, Science for Life Laboratory, Stockholm University, 10691 Stockholm, Sweden; ^b^Institute of Pharmacy and Molecular Biotechnology, Heidelberg University, 69120 Heidelberg, Germany; ^c^Large Scale Structures, Institut Laue-Langevin, 38042 Grenoble, France; ^d^Department of Applied Physics, Science for Life Laboratory, KTH Royal Institute of Technology, 10044Stockholm, Sweden

**Keywords:** ligand-gated ion channel, Cys-loop receptors, small-angle neutron scattering, calcium

## Abstract

Pentameric ligand-gated ion channels (pLGICs) perform electrochemical signal transduction in organisms ranging from bacteria to humans. Among the prokaryotic pLGICs, there is architectural diversity involving N-terminal domains (NTDs) not found in eukaryotic relatives, exemplified by the calcium-sensitive channel (DeCLIC) from a *Desulfofustis* deltaproteobacterium, which has an NTD in addition to the canonical pLGIC structure. Here, we have characterized the structure and dynamics of DeCLIC through cryoelectron microscopy (cryo-EM), small-angle neutron scattering (SANS), and molecular dynamics (MD) simulations. In the presence and absence of calcium, cryo-EM yielded structures with alternative conformations of the calcium-binding site. SANS profiles further revealed conformational diversity at room temperature beyond that observed in static structures, shown through MD to be largely attributable to rigid-body motions of the NTD relative to the protein core, with expanded and asymmetric conformations improving the fit of the SANS data. This work reveals the range of motion available to the DeCLIC NTD and calcium-binding site, expanding the conformational landscape of the pLGIC family. Further, these findings demonstrate the power of combining low-resolution scattering, high-resolution structural, and MD simulation data to elucidate interfacial interactions that are highly conserved in the pLGIC family.

## Significance

The *Desulfofustis* deltaproteobacterium ligand-gated ion channel (DeCLIC) is activated by calcium depletion and, to date, the only member of its family with an additional N-terminal domain for which a structure has been determined. Calcium inhibition is common in this protein family, and the calcium-binding site of DeCLIC shares similarities with other related receptors. We present structures of DeCLIC with and without calcium, demonstrating condition-dependent side-chain rearrangements within the closed state, and show that in the absence of calcium, the site allows a secondary path for sodium to reach the central channel of the protein. Further, solution scattering and simulations reveal the N-terminal domain to be highly dynamic, highlighting asymmetry in these receptors.

Pentameric ligand-gated ion channels (pLGICs) belong to a family of membrane proteins responsible for fast signal transduction, utilized by numerous organisms from bacteria to humans (1). In response to a chemical stimulus, these proteins undergo a conformational change, allowing ion flux across the cell membrane (2). This state-dependent ion permeation is especially important for the proper function of the mammalian nervous system; accordingly, dysfunction in these channels is linked to a variety of neurodegenerative disorders such as epilepsy, hyperekplexia, and Alzheimer’s and Parkinson’s diseases (3). These effects make pLGICs critical targets for a number of therapeutic agents, including anesthetics, benzodiazepines, and neurosteroids (4, 5). A detailed understanding of channel pharmacology and conformational change depends on high-quality structural data, potentially complemented by dynamic information from methods like molecular dynamics (MD) simulations and small-angle scattering (6).

In recent years, advances in X-ray crystallography and cryoelectron microscopy (cryo-EM) (7) have revealed several conserved features of the pLGIC structure (8). All pLGICs share a fivefold homomeric or heteromeric assembly. Each subunit contains a transmembrane domain (TMD) consisting of four helices (M1 to M4) and an extracellular domain (ECD) consisting of ten beta strands (*β*1 to *β*2) and a characteristic Pro- (prokaryotes) or Cys-loop (eukaryotes) (9). Most eukaryotic pLGICs also contain an intracellular domain (ICD) of variable length (2). In contrast, several prokaryotic family members contain an additional amino-terminal domain (NTD) (1, 9) not observed in any eukaryotic homologs. Despite these insights, the details of ion permeation and modulation in this family, and the structure–function relationships of these peripheral domains, remain poorly understood.

**Fig. 1.**
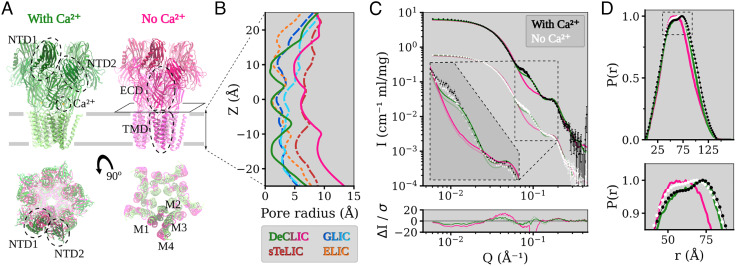
Structure overview of DeCLIC and the fit of the DeCLIC X-ray structures to small-angle neutron scattering data. (*A*) The structure of DeCLIC consists of two beta-rich N-terminal domains (NTD1 and NTD2), a beta-sandwich extracellular domain (ECD) with a calcium-binding site, and a transmembrane domain (TMD) with four helices M1 through M4. X-ray crystallography structures have been determined by Hu et al. (11) in the presence (green) and absence (pink) of calcium, with the absence of calcium leading to a conformation with a more contracted NTD and a more expanded TMD. (*B*) Pore profiles of the TMD region in DeCLIC (green and pink, solid lines) and of the related channels, GLIC (dark and light blue, dashed lines), sTeLIC (dark red, dash-dot line), and ELIC (orange, dotted line). In the presence of calcium, DeCLIC (green line) has a contracted pore similar to ELIC (orange dots) and GLIC at resting conditions (dark blue dashes). In the absence of calcium, DeCLIC (pink line) has a wide pore at the 9’ hydrophobic gate (Z = 0 Å), similar to sTeLIC (dark red dash-dots), and is at the 16’ contraction (Z  ≈  10 Å) similar to GLIC at activating conditions (light blue dashes). (*C*) Small-angle neutron scattering from DeCLIC in the presence (black) and absence (white) of calcium, with predicted curves from the crystal structures in the presence (green) and absence (pink) of calcium. The dataset in the presence of calcium has been offset by a factor of 10 for clarity. The inset shows the data on the same scale zoomed in on *Q* ∈ [0.06, 0.2] Å^−1^, with darker curves fitted to the SANS data with calcium and paler curves fitted to the calcium-free SANS data. The calcium-free crystal structure has poor goodness of fit (χ^2^ > 60) to the SANS data, while the calcium-containing crystal structure gives moderate goodness of fit (χ^2^ of 10.8 to SANS with calcium and χ^2^ of 8.8 to SANS without calcium), deviating from the experimental data mainly for *Q* ∈ [0.06, 0.09] Å^−1^. (*D*) Pair distance distribution of DeCLIC from SANS with (black) and without (white) calcium and from crystal structures with (green) and without (pink) calcium. There is close agreement between the distribution from SANS with calcium and from the calcium-containing crystal structure, while the SANS data collected in the absence of calcium have features similar to those of both crystal structures (zoom box).

Similar to several eukaryotic ion channels (10), the bacterial channel DeCLIC—derived from a *Desulfofustis* deltaproteobacterium—was recently shown to be modulated by circulating calcium ions (11). DeCLIC was crystallized in both the presence and absence of calcium, offering models in apparent closed and open states (Fig. 1*A*). When crystallized in high (150 mM) calcium, DeCLIC bound calcium via several electronegative groups in the ECD periphery, and the permeation pore in this structure was too narrow to conduct ions at multiple points along the TMD (Fig. 1 *A* and *B*).

In the absence of calcium, the pore instead adopted a wide-open *apo* state, notably distinct from related pLGIC structures (Fig. 1 * A* and *B*). The ECD vestibule was also tightly constricted, raising questions about the pathway of ion transit and about the general relevance of these structures in the gating cycle of DeCLIC or other family members. Notably, both structures included a partially resolved NTD. Although not seen in eukaryotic pLGICs, N-terminal domains (NTDs) occur in other ligand-gated channels such as AMPA and NMDA receptors (12), where they can bind ligands and have allosteric effects (13). In the case of DeCLIC, the NTD was found to influence gating kinetics, with each subunit containing two jelly-roll lobes (NTD1 and NTD2) packed against the peripheral ECD (Fig. 1*A*). However, details about functional and structural relevance of this domain remain unclear (11).

Here, we sought to understand the mechanisms of ion binding and permeation, the physiological relevance of resolved states, and the dynamic behavior of domains in DeCLIC, using three complementary biophysical methods. In particular, we applied small-angle neutron scattering (SANS) to obtain low-resolution information on the room temperature solution structure, single-particle cryo-EM to determine higher-resolution structural information that avoids crystal contacts, and MD simulations for observing dynamics and conformational sampling at the atomic level. Our data support a dominant population of closed channels in both the presence and absence of calcium and a mechanism for calcium block via a peripheral site at the ECD–subunit interface. We further find that the spontaneous translation of the NTD lobes, especially NTD1, helps describe some features of our SANS data and supports a new detailed model of pLGIC conformational diversity.

## Results

### Small-Angle Neutron Scattering Reveals Deviations from Static Structures.

To investigate solution-phase structural properties of DeCLIC in the presence and absence of inhibitory calcium, we collected SANS data in either 10 mM CaCl_2_ (Ca^2+^) or 10 mM EDTA (*apo*) conditions. To optimize for homogeneous monodisperse samples, data were collected from a single peak from an inline size exclusion column (6). As calculated by Guinier analysis, the sample radius of gyration (R_g_) was slightly larger under Ca^2+^ conditions than *apo* conditions (52.0 ± 0.2 Å compared to 51.6 ± 0.2 Å). Similarly, R_g_ calculated from X ray structures in the presence and absence of Ca^2+^ (49.5 Å and 48.2 Å, respectively) was also larger in the presence of Ca^2+^ (*SI Appendix*, Fig. S1 and Tables S1 and S2). Molecular weight estimates in Ca^2+^ and *apo* conditions (348 kD and 318 kD, respectively) were within 10% of that predicted from the amino acid sequence (342 kD), which is within the expected uncertainty arising from concentration determination. Accordingly, we proceeded to compare fits of the full scattering profiles with curves predicted from past X-ray structures.

Scattering profiles under Ca^2+^ and *apo* conditions were similar, and both were better described by the closed X-ray structure (χ^2^ = 10.8 for Ca^2+^, χ^2^ = 8.8 for *apo*) than by the open structure (χ^2^ > 60 for data collected under either condition) (Fig. 1*C*). Deviation from the closed-state curve was primarily evident for a feature around *Q* ∈ [0.06, 0.09] Å^−1^ (Fig. 1*C*, dashed inset). As real-space distances relate to momentum transfer by *d* = 2*π*/Q, this feature corresponds to a range of 70 to 105 Å, a relatively long range compared to the better-matched feature around *Q* ∈ [0.10, 0.20] Å^−1^. Overall, the distribution of pairwise distances was well described by the closed X-ray structure (Fig. 1*D*), though it did show a slight underestimation of the prevalence of distances > 100 Å (*SI Appendix*, Fig. S2*A*) that could indicate the presence of more expanded conformations. The pairwise distance distribution from SANS data under *apo* conditions deviated modestly from both calcium-SANS data and the closed structure, with an increased contribution of shorter distances approaching the distribution function for the open structure. Thus, SANS curves under both Ca^2+^ and *apo* conditions were consistent with predominantly closed populations of channels, with some deviations at relatively long pairwise distances, and in the overall distribution of distances in the *apo* state.

### Differential Closed cryo-EM Structures in the Presence and Absence of Calcium.

**Fig. 2.**
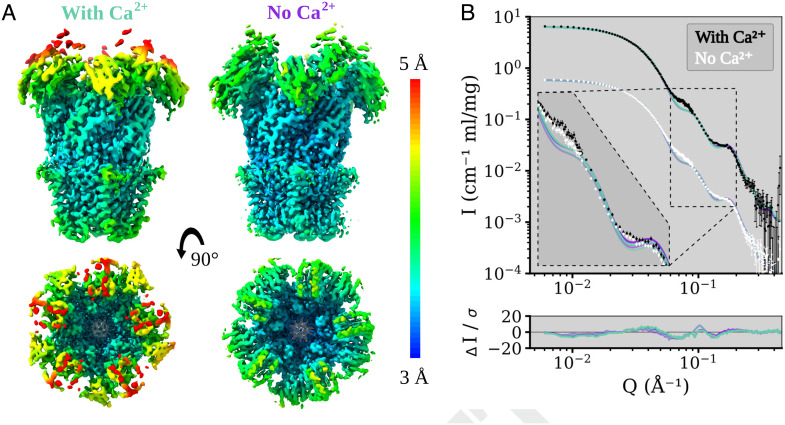
Local resolution coloring of the DeCLIC Cryo-EM reconstructions in the presence and absence of calcium and their agreement with the SANS data. (*A*) Cryo-EM densities of DeCLIC in the presence of 10 mM Ca^2+^ (*Left*), and from a Ca^2+^ chelated condition (*Right*). The densities were resolved to over all resolutions of 3.5 Å (condition with Ca^2+^) and 3.2 Å (condition with no Ca^2+^) respectively. Density is colored by local resolution according to the scale bar to the right. The quality is worse in the NTD region of the protein compared to that of the TMD or ECD. (*B*) Fits of model spectra calculated from the two cryo-EM structures in the presence (aquamarine) and absence (purple) of Ca^2+^ to SANS data collected with Ca^2+^ (black) and with no Ca^2+^ (white). The structures yield similar goodness of fit, χ^2^∈[11.2, 12.1]. SANS data with Ca^2+^ are offset by a factor of 10 for easier visualization. The inset shows the data on the same scale zoomed on *Q* ∈ [0.06, 0.2] Å^−1^, with darker curves fitted to the with-calcium SANS data and paler curves fitted to the calcium-free SANS data.

To complement our SANS curves, we next collected cryo-EM data for DeCLIC in the presence and absence of 10 mM Ca^2+^, with the aim of sampling the channel’s conformational space without crystal contacts or high-mM calcium. Each dataset yielded a single C5-symmetric reconstruction, with comparable resolution (3.5 Å and 3.2 Å in the presence and absence of calcium, respectively) (Fig. 2*A*, *SI Appendix*, Figs. S3 to S5 and Table S3). Classification was also attempted without symmetry but yielded only lower-resolution densities. For both conditions, the resolution was higher in the TMD and ECD but worsened to ≥4.5 Å in the NTD (Fig. 2*A*). Although the backbone and majority of side chains were clearly resolved in the TMD and ECD (*SI Appendix*, Fig. S6), some residues could not be built in NTD1 and the NTD1-NTD2 loop (*SI Appendix*, Table S4). The quality of the NTD was moderately better in the absence of calcium, with more residues resolved in both lobes (*SI Appendix*, Table S4).

Independent models were built manually in the two conditions. The greatest difference between them was around each ECD–subunit interface, particularly involving the β1 to β2 loop and loop F, as detailed in the next section. Otherwise, the two models were largely superimposable (RMSD ≤ 0.63 Å) particularly in the TMD, with similarly closed pores. Indeed, the pore profiles of both models included hydrophobic constrictions to ≤1 Åradius at both L554 (9’) and F561 (16’) in the pore-lining M2 helices (*SI Appendix*, Fig. S7*A*), consistent with features of previous X-ray structures (11). The ECD vestibule in both models featured an additional constriction to ∼5 Å radius, defined primarily by residue N405 in the β4 to β5 loop (*SI Appendix*, Fig. S7*B*). A weaker density in this vestibule could be attributed to an alternative, inward-facing, rotamer of residue W407. Interestingly, W407 was previously reported to orient into the ECD vestibule upon crystallization in the absence of Ca^2+^, leading to an even tighter constriction potentially incompatible with ion conduction via a linear ECD pathway (11).

Next, we compared theoretical curves predicted from our cryo-EM models to the SANS data described above (Fig. 2*B*). Similar fits were obtained for curves based on either the calcium-bound (χ^2^ = 12.1 for Ca^2+^-SANS, χ^2^ = 11.3 for *apo*-SANS) or calcium-free cryo-EM models (χ^2^ = 11.2 for Ca^2+^, χ^2^ = 11.7 for *apo*). Pairwise distribution functions for the two cryo-EM models were also comparable (*SI Appendix*, Fig. S2*A*). However, similar to the closed X-ray structure, a feature in the range *Q* ∈ [0.06, 0.09] Å^−1^ was poorly fit by both cryo-EM models (Fig. 2*B*, *Inset*). Thus, cryo-EM data broadly confirmed our SANS curves in substantiating a predominant population of closed channels, while leaving some features of the scattering data unexplained.

### Ion Interactions at the Calcium Site Revealed by cryo-EM and Molecular Dynamics.

**Fig. 3.**
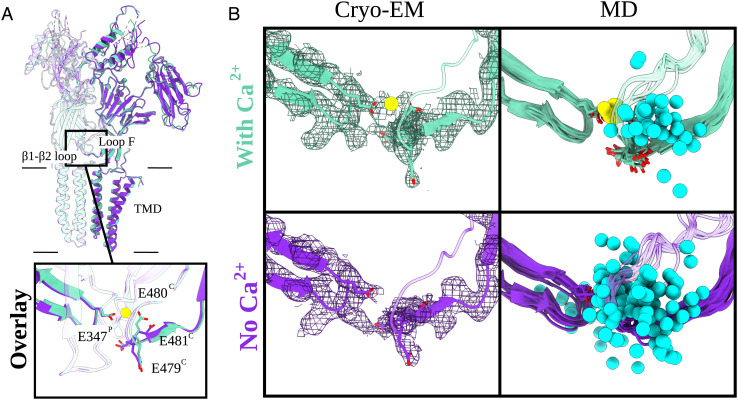
Arrangement of the calcium-binding residues elucidated by cryo-EM and MD. (*A*) Overlay of two adjacent subunits of DeCLIC cryo-EM structures with and without Ca^2+^ (aquamarine and purple, respectively). Protein domains and main Ca^2+^-binding motifs are shown. Those include the transmembrane domain (TMD), extracellular domain (ECD), and two amino-terminal domains (NTD1 and NTD2) as well as the β1 to β2 loop from the principal subunit (P) and loop F from the complementary subunit (C). The boxed-in view (*Bottom*) shows the relevant side chains (sticks, colored by heteroatom). The Ca^2+^ ion present in the Ca^2+^ dataset (aquamarine) is colored in yellow. (*B*) Cryo-EM panels (*Left*) represent the cryo-EM structures and their corresponding densities (mesh at α  =  0.012 to 0.015). Relevant side chains around the Ca^2+^-binding site [loop F (C), β1 to β2 loop (P)] are displayed as sticks (heteroatom coloring). The density for the Ca^2+^-binding residues as well as for the Ca^2+^ ion (in the with-Ca^2+^ condition) is well resolved. MD panels (*Right*) illustrate eleven individual conformations (one snapshot for every 100 ns) from 1-μs long MD simulations of the cryo-EM models. The relevant Ca^2+^ residues are displayed as sticks (colored by heteroatom). In cyan, we have Na^+^ ions (sphere, trajectories generated for every 10 ns) and Ca^2+^ ion in yellow. In the no-Ca^2+^ condition, the Na^+^ ions are free to enter the cavity which is occupied by Ca^2+^ in the with-Ca^2+^ condition.

Although cryo-EM models of DeCLIC in the presence and absence of calcium were similar in apparent conductance states, they exhibited notable differences at each ECD–subunit interface. The quality of the densities in this region allowed building of the complete backbone and side chains of the β1 to β2 loop on the principal side of each interface and of loop F on the complementary side (Fig. 3 *A* and *B*). The dataset with calcium also featured a small nonprotein density between these loops (Fig. 3*B*). Because this density was absent in the calcium-free dataset, and located near several acidic side chains (E347, E479, E480, and E481), it was interpreted as a Ca^2+^ ion. In particular, carboxylate atoms of residues E347 and E480 (Fig. 3*B*, *SI Appendix*, Fig. S8 *B* and *C*) were positioned < 3 Å from the modeled ion, primed to coordinate it from both sides (Fig. 3*B* and *SI Appendix*, Fig. S8 *B* and *C*). In the calcium-free model, this ion density was absent, and E480 adopted a different rotamer facing away from the subunit interface (Fig. 3 *A* and *B*). Accordingly, the distance between E347 and E480 increased from < 6 Å to > 8 Å.

To confirm the stability of this putative calcium-binding site, we performed quadruplicate 1-μs all-atom MD simulations of each cryo-EM model, inserted in a lipid bilayer and solvated in water and sodium chloride. These simulations relaxed to stable conformational ensembles within 200 ns in the ECD and TMD (C*α* RMSD < 2 Å, *SI Appendix*, Fig. S9 *D* and *E*); the NTD was substantially more variable, as described in subsequent sections. During simulations of the model with calcium, the distance between putative calcium-coordinating residues E347 and E480 remained < 7 Å (*SI Appendix*, Fig. S8 *B* and *C*, *Center*). Proximal residues E479 and E481 were also stably oriented within 13 Å of E347, possibly contributing to the negative electrostatic environment of the binding site (*SI Appendix*, Fig. S8 *B* and *C*, *Left and Right*). Moreover, all five calcium ions remained bound in all simulations, and no other ions were seen to transit the interface between subunits (Fig. 3*B*). Conversely, in simulations of the cryo-EM model without calcium, E480 sampled various orientations up to 10 Å from E347 (*SI Appendix*, Fig. S8 *B* and *C*, *Center*). Residues in loop F also fluctuated more in simulations of the calcium-free versus calcium-bound cryo-EM models (*SI Appendix*, Fig. S10). Interestingly, the unoccupied calcium site at each subunit interface was frequently filled by sodium ions, in some cases transiting to or from the ECD vestibule (Fig. 3*B* and Movie S1).

To assess the possible influence of experimental conditions and models obtained with different techniques, we also ran simulations of the previously reported closed X-ray structure (11). This structure was chosen due to its overall similarity to both our cryo-EM models and was simulated in the presence and absence of calcium ions at the proposed binding site. In the X-ray structure, calcium was primarily coordinated between E347 and E479, rather than E480 (*SI Appendix*, Fig. S8*A*). Nonetheless—echoing simulations of our cryo-EM models—the distance between coordinating residues remained below 6 Å with calcium present and increased to more than 10 Å with calcium removed (*SI Appendix*, Fig. S8 *B* and *C*, *Left*). Removing calcium from the X-ray structure also increased fluctuations in loop F (*SI Appendix*, Fig. S10). Thus, structural and simulations data supported a stable binding site for calcium at the β1-β2/loop F subunit interface, with unbinding mobilizing loop F and allowing transit of monovalent ions.

### Simulations Demonstrate Relative Instability of Open Versus Closed States.

**Fig. 4.**
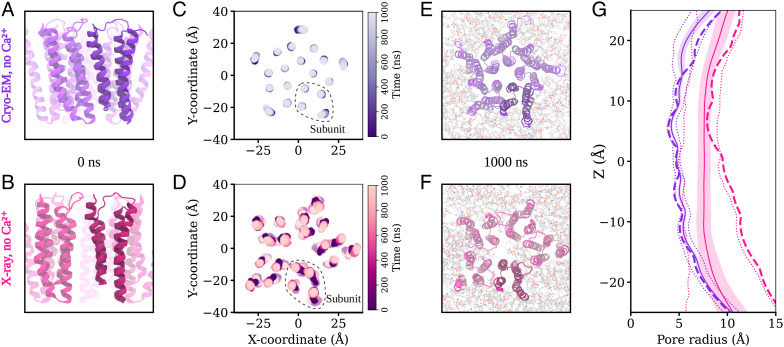
Behavior of the transmembrane domain in molecular dynamics simulations. (*A* and *B*) View of the transmembrane region of DeCLIC, seen from the membrane plane, prior to MD simulation in the calcium-free cryo-EM structure and in the calcium-free X-ray structure. (*C* and *D*) Position of the center of mass of each transmembrane helix in the bilayer plane through 1,000 ns of MD simulations and four replicas. For the calcium-free cryo-EM structure, the helices of the transmembrane region remain close to their positions in the structure, while the transmembrane domain in simulation of the X-ray structure without calcium undergoes a conformational change to an asymmetric arrangement of the subunits. (*E* and *F*) Transmembrane region seen from the extracellular side at the end of the simulations. The cryo-EM structure without calcium has retained a symmetric arrangement, while the X-ray structure without calcium has become asymmetric, with lipids penetrating to the pore in one simulation replica. (*G*) Pore profiles tracing the protein backbone for the calcium-free cryo-EM (purple) and X-ray (pink) systems. Dashed lines show the profile for the starting structure, solid lines the simulation average with SD in colored fill, and dotted lines minimum and maximal values during the simulations. There is little variation in the pore profile of the cryo-EM system during the simulations, while the wide lower part of the pore in the calcium-free X-ray structure contracts during the simulations.

Simulations of closed DeCLIC structures determined by either cryo-EM or X-ray crystallography were notably stable in the TMD (Fig. 4 *A* and *G* and *SI Appendix*, Fig. S7*A*), with only minor deviations in the peripheral M4 helices (Fig. 4*C*; *SI Appendix*, Fig. S11). Although no open state was evident in our cryo-EM data, we also sought to compare the dynamics of open DeCLIC by simulating a previously reported X-ray structure with a wide-open pore (11). However, all simulations of this system exhibited substantial asymmetric deviations in TMD architecture, largely associated with the sizable gaps located between subunits in the initial structure (Fig. 4 *B* and *D*). Placing lipids into a partially resolved upper-leaflet electron density, or into plausible interfacial positions in both the upper and lower leaflets, failed to prevent deformation of the TMD (*SI Appendix*, Fig. S11). With or without manually placed lipids, lipids frequently penetrated the TMD–subunit interface, in some cases entering the channel pore (*SI Appendix*, Fig. S12). The associated deformations were further associated with variable pore collapse (Fig. 4*G* and *SI Appendix*, Fig. S7*A*), rendering this system unsuitable for open-state simulations. Thus, although closed-pore structures were largely validated by MD, at least in our hands, the wide-open X-ray state did not appear to be stable under any simulation conditions attempted.

### Simulations of Alternative NTD States Refine Fit to SANS Data.

**Fig. 5.**
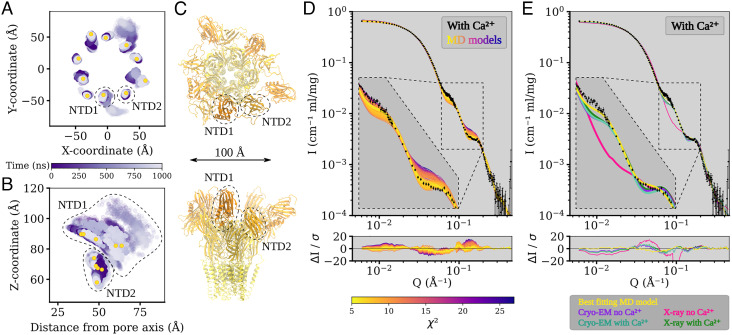
The N-terminal domains of DeCLIC are highly dynamic, and an asymmetric arrangement thereof with a mix of contracted and extended NTD positions yields the best fit to the SANS data. (*A* and *B*) Position of the center of mass of each NTD lobe over time in four MD simulations, showing their positions in the XY-plane (*A*) and their Z-coordinate as a function of distance from the pore axis (*B*). NTD1 is highly mobile, sampling positions further out, lower down, and higher up than the starting conformation. NTD2 has less mobility, mainly sampling positions along Z. Yellow dots show the position of the NTD lobes in *C*. (*C*) Snapshot of the MD simulation frame yielding the best fit to the with-calcium SANS data (χ^2^ of 5.2), seen from the extracellular side (*Top*) and from the plane of the membrane (*Bottom*). The protein has adopted an asymmetric conformation with three NTD1 domains in positions similar to the determined structures and two NTD1 lobes further out and down. The snapshot is from the simulations started from the no-Ca^2+^ cryo-EM structure. (*D*) Fits of snapshots from simulations of the no-Ca^2+^ cryo-EM structure and the error-weighted residual between the models and the scattering profile. The simulations yield theoretical distributions around the experimental scattering curve, containing models with better fit (best χ^2^ of 5.2) to the data than the experimental structure from which the simulations were launched (χ^2^ of 11.2). Inset shows *Q* ∈ [0.06, 0.2] Å^−1^. (*E*) Comparison between the best-fitting model from simulations (yellow), structures of closed-like DeCLIC (purple, aquamarine, and green; note that curves overlap), and the X-ray no-Ca^2+^ structure (pink). As seen in the inset showing *Q* ∈ [0.06, 0.2] Å^−1^, the simulation model has the best fit to both features in the scattering profile.

In contrast to the ECD and TMD, in all simulation systems, the NTD was notably dynamic, moving substantially relative to its starting conformation (Fig. 5 *A* and *B* and *SI Appendix*, Fig. S13 to S15). Indeed, when aligned on the ECD–TMD scaffold, average C_α_ RMSDs for the NTD1 and NTD2 lobes reached over 10 Å and 6 Å, respectively (*SI Appendix*, Fig. S9 *B* and *C*). This variability could largely be attributed to rigid-body motions of individual lobes, as deviations within each lobe remained at 3 Å when it was aligned on itself (*SI Appendix*, Fig. S9 *B* and *C*). The NTD1 lobes sampled a range of positions, often translated outward from the pore (z-)axis and down toward the membrane; NTD2 primarily translated up or down along z (Fig. 5 *A* and *B*; *SI Appendix*, Fig. S13 to S15). In several cases, these transitions appeared to be reversible, with lobes returning to positions near their starting poses within 1 μs (*SI Appendix*, Figs. S14 to S15). As in previous work (6), we further used principal component (PC) analysis to characterize major conformational changes sampled in DeCLIC simulations. Here, we identified relevant PCs on the basis of simulation snapshots rather than experimental structures, to more thoroughly sample conformations, particularly of the NTD. The first two PCs captured motions in the NTD and TMD (*SI Appendix*, Fig. S16*A*); notably, projection along these PCs separated snapshots into distinct clusters of closed- versus open-state simulations (*SI Appendix*, Fig. S16*B*).

All simulations included snapshots that improved goodness of fit to our Ca^2+^-SANS data, relative to our static cryo-EM or X-ray structures (Fig. 5*D* and *SI Appendix*, Figs. S17 to S18). Better goodness of fit to the feature at *Q* ∈ [0.06, 0.09] Å^−1^ generally corresponded to alternative conformations of the NTD, particularly asymmetric arrangements of NTD1. For instance, in the overall best-matching snapshot (χ^2^ = 5.2; Fig. 5 *C* and *E*), two NTD1 lobes were translated outward from the z-axis (i.e., the pore axis) and down toward the membrane, relative to the starting model (cryo-EM model without calcium, χ^2^ = 11.2). Other snapshots produced even better goodness of fit to the *Q* ∈ [0.06, 0.09] Å^−1^ feature, with more dramatic translation of the NTD1 lobes (*SI Appendix*, Fig. S19). However, SANS curves calculated from these models were poor matches for the *Q* ∈ [0.11, 0.19] Å^−1^ feature that corresponds to shorter interatomic distances, resulting in worse goodness of fit overall. Projected onto the first two components in PC space described above, snapshots with better goodness of fit to calcium-SANS data clustered with closed-state simulations (*SI Appendix*, Fig. S16*C*). Indeed, the best-fit snapshot projected to an approximate centroid of the closed-state cluster, suggesting that it was a representative state. Still, snapshots with relatively high goodness of fit projected to the full range of values along both PC1 and PC2 in this cluster, indicating that general NTD flexibility may be as important a feature of the SANS population as any particular conformation.

Interestingly, simulations did not improve goodness of fit to our *apo*-SANS data: the best simulation frame (χ^2^ = 9.4) was comparable to the best static model (X-ray model with calcium, χ^2^ = 8.8) (*SI Appendix*, Figs. S17 to S18), and while it did improve correspondence to long pairwise distances, it did not match the distribution of medium distances (*SI Appendix*, Fig. S2*B*). In PC space, it can clearly be seen that simulation snapshots generally fit less well to SANS data collected in the absence versus presence of calcium and with less distinctive clustering with closed versus open-state simulations (*SI Appendix*, Fig. S16*D*). Thus, in the presence of calcium, DeCLIC’s solution state was best approximated by an asymmetric modification of the cryo-EM structure, including some NTD1 lobes translated “out-and-down.” In the absence of calcium, no single structure or snapshot provided as strong a prediction of the SANS profile, possibly implicating the presence of an alternative state.

## Discussion

**Fig. 6.**
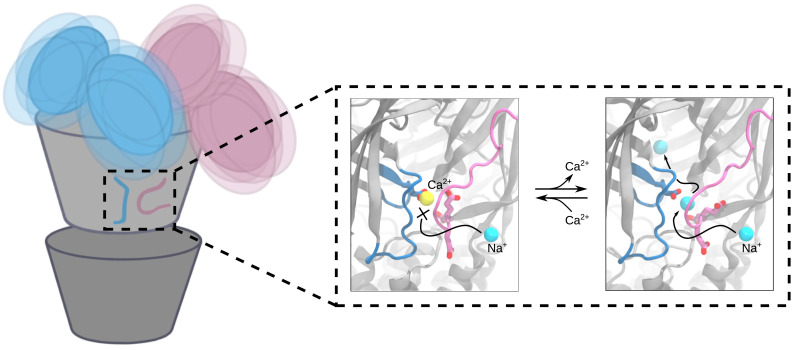
Conformational variability and proposed calcium-binding site behavior in closed DeCLIC. The positions of the N-terminal domains of DeCLIC fluctuate around the core of the protein, sampling a wide range of conformations where the average can be described by an asymmetric conformation with a mix of compact and extended NTD positions. In the calcium-binding site, bound calcium blocks sodium access to the site. Following calcium depletion, sodium can enter the central pore through the calcium-binding site. The calcium-binding site is thus proposed to act as a supplementary way for sodium ions to reach the ion conduction pathway, with the three consecutive glutamates on loop F shepherding sodium from outside the protein, through the calcium-binding site, and into the extracellular part of the ion channel pore.

The combination of SANS, cryo-EM, and MD has enabled us to describe a plausible mechanism for extracellular calcium modulation and ion permeation and of dynamic NTD states corresponding to solution-phase conditions (Fig. 6). When calcium is present, it is tightly bound at an interfacial site in the canonical ECD, where it blocks ions from transiting between the extracellular vestibule and external medium via this interface (Fig. 6, *Center*). In the absence of calcium, monovalent cations such as sodium can traverse this interfacial site, possibly facilitated by the electronegative cluster of glutamate residues on the principal *β*1 to *β*2 loop and complementary loop F (Fig. 6, *Right*). Notably, ion paths to the transmembrane pore through electronegative fenestrations rather than the linear axis of the ECD have also been reported in both nicotinic (14) and GABA_A_ receptors (15–17), including acidic residues on the *β*1 to *β*2 loop, Cys-loop, and loop F.

Interfacial fenestrations may be particularly relevant to ion permeation in DeCLIC. First, all known DeCLIC structures contain a constriction in the outer-ECD vestibule (*SI Appendix*, Fig. S7), which could reduce transit to or from the transmembrane channel via a linear pathway. Indeed, in the X-ray structure previously reported in the absence of calcium, (11) the linear path was even tighter at this outer-ECD point, a relative contraction that was maintained throughout our MD simulations (*SI Appendix*, Fig. S7). Given that this structure was determined under activating conditions—albeit with instabilities possibly attributable to crystal packing—the peripheral fenestrations might provide pathways equally or more favorable to ion permeation than the linear pathway in the open state. Permeation of sodium via a highly electronegative fenestration is also consistent with the anticipated cation selectivity of DeCLIC. Although instabilities in the X-ray open state precluded definitive characterization of permeation through the full-length channel, DeCLIC was previously classified as cation-selective (11), given the presence of two rings of acidic residues lining the cytoplasmic mouth of the pore. An equivalent selectivity filter has been observed in all known cation-selective eukaryotic and prokaryotic pLGICs (18). Further exploration of experimental conditions capable of characterizing a stable open state of DeCLIC will be required to clearly validate cation selectivity in this system.

**Fig. 7.**
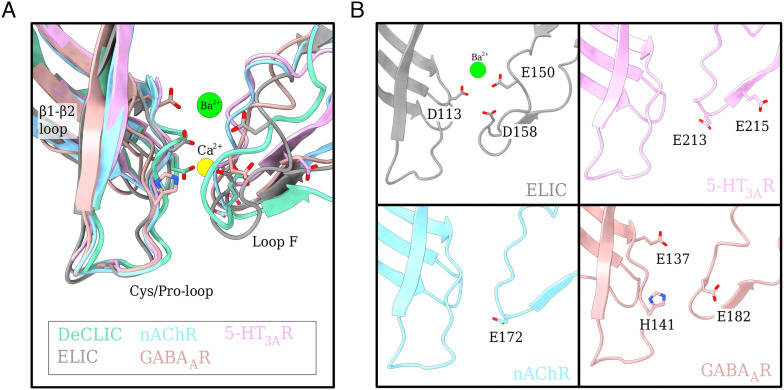
Charged residues at the subunit interface present in other family members. (*A*) Overlay of the subunit interface (loop F, Cys/Pro-loop, and *β*1 to *β*2 loop) from DeCLIC with other family members, nAChR (sky blue), ELIC (gray), 5-HT_3A_R (pink), and GABA_A_R (salmon) with Ca^2+^ ion in yellow and Ba^2+^ in green. The important charged residues are displayed as sticks (colored by heteroatom). (*B*) Display of each family member from (*A*) individually for clearer representation with relevant charged residues displayed as sticks and colored by heteroatom.

Tight divalent-cation binding at interfacial fenestrations is further reminiscent of calcium modulation in the larger family of pLGICs. In this context, calcium has been shown to inhibit both the bacterial ELIC and eukaryotic serotonin receptor (5-HT_3A_R) (10, 19) and to potentiate the neuronal *α*7-nicotinic acetylcholine receptor (nAChR) (20, 21). Divalent zinc ions have also been shown to inhibit GABA_A_ receptors (22) and to exert bimodal effects on glycine (GlyR), nicotinic, and 5-HT_3A_ receptors (23–25). Although a variety of specific residues have been implicated in these effects, most are located at the ECD subunit interface, involving loop F as well as the *β*1 to *β*2 and Cys/Pro-loops (Fig. 7) (10). Acidic loop F residues E150 and D158 in ELIC (10), E213, and E215 in 5-HT_3A_R (26), E172 in *α*7-nicotinic (27, 28), and E182 in GABA_A_ receptors (29) have been shown to be particularly important, suggesting a common strategy for divalent ion modulation. Interestingly, the previous calcium-free X-ray structure of DeCLIC exhibited not only side-chain rearrangements as seen in our calcium-free cryo-EM structure but also dramatic remodeling of the *β*1 to *β*2 loop and loop F (11). Such changes could represent a state further along the opening-transition path, shedding potential light on gating and modulation. Still, relative instability of the calcium-free X-ray structure, particularly in the NTD and TMD (*SI Appendix*, Fig. S9), indicates that this model may be subject to crystallization artifacts and precludes definitive modeling of mechanistic transitions.

Aside from the ECD calcium site, structures in the presence and absence of calcium were largely superimposable, including a closed pore. This observation was initially surprising, given previous evidence for channel opening upon calcium depletion, from both electrophysiology and crystallography (11). It is possible that only a minority population of channels is open even under maximally conducting conditions. Alternatively, DeCLIC may require an additional signal aside from calcium depletion to open substantially under SANS or cryo-EM conditions. We cannot rule out the effects of detergent in biasing the landscape toward closure, though equivalent solubilization was apparently permissive of wide opening in previous crystallographic work (11). It is also theoretically possible that a fraction of open channels exist in the data but that particle classification failed to identify them as a substantial discrete population of channels, as has previously been reported for the related bacterial channel GLIC (30).

Pairwise distance distributions from SANS data did indicate the promotion of an alternative state in the absence of calcium, consistent with a limited population similar to the open X ray structure. Similarly, the radius of gyration was moderately reduced by calcium removal (*SI Appendix*, Table S1 and *Multiple Methods*), indicating a contribution of relatively compact NTD states under *apo* conditions. Further rigidification of the NTD—for example, via crystal contacts—could stabilize an open state of the channel, otherwise underrepresented in solution. However, MD simulations were capable of improving goodness of fit to the SANS profile in calcium but not *apo* conditions, indicating that the available structures—closed and open—did not spontaneously sample conformations representative of the *apo* time and population average. We have previously reported improved SANS goodness of fits to specific pLGIC states by a linear combination of closed and open structures (6); however, such an approach relies on trusted models of all conformations contributing to the population. Instead, here, our simulations failed to support the open X-ray structure as a stable state, leaving open questions as to the physiologically relevant conductive state.

Indeed, our present data do not definitively distinguish whether the interfacial calcium site plays a functionally relevant or primarily structural role. The conversion of the interfacial site from rigid calcium coordination to dynamic sodium permeation suggests a role for calcium in blocking at least one type of ion pathway but cannot rule out additional or predominant allosteric effects of calcium on DeCLIC function. SANS data indicated a different population in the presence versus absence of calcium, suggesting an allosteric effect on the conformational landscape; it is possible that an open state of DeCLIC is relatively stabilized by calcium depletion, although conditions of our cryo-EM experiment failed to sufficiently favor this state to clearly resolve representative particles. Either way, it is possible that additional calcium interaction sites—perhaps localized in less well-resolved domains—could influence the landscape more strongly than the interfacial site identified here. Further insight into the functional relevance of the interfacial site to calcium modulation in DeCLIC and its evolutionary neighbors will likely depend on further structure–function analysis.

Although both X-ray and cryo-EM structures substantiated a symmetrical, compact predominant state of the DeCLIC NTD, data from all three methods here also supported substantial mobility in this domain (Fig. 6, *Left*). Flexibility in the NTD is consistent with its relatively low resolution and with motions directly observed in MD simulations, as well as the ability of MD results to describe solution-phase SANS data better than static structures. Because SANS profiles reflect the time- and population-average conformation, this model could either represent the most probable conformation of an individual protein or an average of a mixture of states. Simulation results offer more specific predictions that asymmetric arrangements of the NTD lobes are common and rapidly interchangeable. The range of NTD motion allowed this domain to sample a volume around the protein considerably larger than originally predicted by structural data, possibly facilitating encounters between the NTD and yet-unknown interaction partners in the periplasmic space. Thus, alongside insights into ion interactions and pore stability, this work highlights conformational features and intrinsic challenges of determining structures of proteins with differentially dynamic domains.

## Materials and Methods

### DeCLIC Expression and Purification.

Expression and purification of DeCLIC-MBP was adapted from the protocol published by Hu et al. (11). In short, C43(DE3) *Escherichia coli* transformed with DeCLIC-MBP in pET-20b were cultured overnight at 37 °C. Cells were inoculated 1:100 into 2xYT media with 100 μg/mL ampicillin, grown at 37 °C to OD_600_ = 0.8, induced with 100 μM isopropyl-β-D-1-thiogalactopyranoside (IPTG), and shaken overnight at room temperature. Cells were resuspended in buffer A (300 mM NaCl, 20 mM Tris-HCl pH 7.4) supplemented with 1 mg/mL lysozyme, 20 μg/mL DNase I, 5 mM MgCl_2_, and protease inhibitors. Membranes were harvested from cell pellets by sonication and ultracentrifugation and then immediately solubilized in 2% n-dodecyl-β-D-maltoside (DDM). Fusion proteins were purified in batch by amylose affinity (NEB), eluting in buffer B (buffer A with 0.02% DDM) with 2–20 mM maltose, then further purified by size exclusion chromatography (SEC) in buffer B. After overnight thrombin digestion, DeCLIC was isolated from its fusion partner with a final size exclusion run and concentrated to 3 to 5 mg/mL by centrifugation.

### Small-Angle Neutron Scattering.

SEC-SANS experiments (31) were performed at the D22 beamline of Institute Laue–Langevin, using a paused flow approach (6). In short, the protein sample was loaded on a Superdex 200 Increase 10/300 gel filtration column in line with the SANS measurement (32, 33), exchanging the sample to the D_2_O buffer environment and match-out deuterated DDM (d-DDM) (34) prior to the protein reaching the SANS measuring cell. Upon peak detection by UV–vis absorbance at 280 nm, the flow was slowed to 0.01 ml/min, and at the peak max, the flow was paused. Two detector distances were used to measure the scattered intensity as a function of momentum transfer Q, 2.8 m/2.8 m during the run, and 8 m/8 m while paused. The definition of Q used was Q = (4 π/λ)sin(*θ*), where 2*θ* is the scattering angle and λ is the wavelength. Measurements were performed under two buffer conditions, with calcium (D_2_O, 150 mM NaCl, 20 mM Tris⋅HCl, 10 mM CaCl_2_, and 0.5 mM d-DDM) and without calcium (D_2_O, 150 mM NaCl, 20 mM Tris⋅HCl, 10 mM EDTA, and 0.5 mM d-DDM). Further sample details and collection parameters are available in *SI Appendix*, Tables S5 and S6.

Data reduction was performed using GRASP version 9.04 (35), correcting for the empty cuvette and background, scaling by transmission and thickness, scaling to absolute intensity by direct flux measurement, and subtracting the buffer contribution using data from a dedicated buffer measurement. Concentration normalization was performed by dividing the scattering intensity with the average protein concentration during the measurement, which was calculated from the corecorded chromatogram and the extinction coefficient calculated from the amino acid sequence using ProtParam (36). Data from the two detector distances were merged using data collected at 8 m up to 0.09 Å^−1^ and for higher Q-values data collected at 2.8 m. A small additional constant was subtracted as a final adjustment of the background.

Guinier analysis was used to calculate I(0) and radius of gyration (R_g_), and the molecular weight was estimated from I(0) (*SI Appendix*, Table S1). The excess scattering length density was calculated using the scattering length density of DeCLIC and D_2_O, (*SI Appendix*, Table S5), accounting for hydrogen-deuterium exchange of labile hydrogens in DeCLIC by calculating the expected degree of exchange after 100 min (approximately the time from the start of a run until the peak max) at pH 7.5 using PSX (Protein-Solvent Exchange) (37). As DeCLIC is a transmembrane protein, the labile hydrogens in the transmembrane region can be expected to be shielded from exchange, which was accounted for by considering hydrogens within the transmembrane region, as defined by the Positioning of Proteins in Membranes (PPM) webserver (38), as ^1^H. The partial specific volume was estimated using the volume calculated by ^3^V (39) for the X-ray structure with calcium and the molecular weight expected from the amino acid sequence. Pair distance distributions from the experimental SANS were calculated using BayesApp (40)—available at https://somo.chem.utk.edu/bayesapp/—and compared with the distribution calculated with CaPP (Calculating Pair distance distribution functions for Proteins) (41) from all atom models. Theoretical scattering curves from all atom models (cryo-EM structures, X-ray structures, and MD snapshots) and their fits to the experimental scattering profiles were calculated using PEPSI-SANS (42). A summary of equations and software used is available in *SI Appendix*, Table S7.

### Cryo-EM Sample Preparation and Data Acquisition.

For each of the experimental conditions, 3 μl of DeCLIC sample was applied to a glow-discharged quantifoil 1.2/1.3 Cu 300 mesh grid (Quantifoil Micro Tools), which was then blotted for 1.5 s and plunge-frozen into liquid ethane using an FEI Vitrobot Mark IV. Micrographs were collected on an FEI Titan Krios 300 kV microscope with a post energy filter Gatan K2-Summit direct detector camera. Movies were collected at nominal 165,000× magnification, equivalent to a pixel spacing of 0.82 Å. A total dose of 40 e^−^/Å^2^ was used to collect 40 frames over 6 s, using a nominal defocus range covering −1.4 to −3.2 μm (for the no-Ca^2+^ dataset) and −2.0 to −3.6 μm (for the Ca^2+^-present dataset) in steps of 0.2 μm. There is a slight difference in the defocus values of the collected datasets, which was selected to optimize the signal-to-noise ratio and contrast of the particles for collection on two different microscopes.

### Image Processing.

Motion correction was carried out with MotionCor2 (43). All subsequent processing was performed through the RELION 3.1 pipeline (44). Defocus was estimated from the motion-corrected micrographs using CtfFind4 (45). Following manual picking, initial two dimensional (2D) classification was performed to generate references for autopicking. Particles were extracted after autopicking and binned before the initial reference was generated. The aberrant particles were removed from the dataset through multiple rounds of 2D and three dimensional (3D) classification as well as 3D autorefinement. Per-particle CTF parameters were estimated from the resulting reconstruction using RELION 3.1. Global beam-tilt was estimated from the micrographs and correction applied. The final 3D auto-refinement was performed using a soft mask, followed by postprocessing with the same mask. Local resolution was estimated using the RELION implementation. Postprocessed densities were improved using ResolveCryoEM, a part of the PHENIX package (release 1.18 and later) (46) based on maximum-likelihood density modification (47). Densities from both RELION postprocessing and ResolveCryoEM were used for building; figures show output from RELION post-processed (Fig. 3 and *SI Appendix*, Fig. S6).

### Model Building.

Models were built starting from a monomer of an X-ray structure determined at pH 7 in the presence of Ca^2+^ (PDB ID: 6V4S (11), chain A), where the missing residues in the NTD region were built by MODELLER (48). PHENIX 1.19.2-4158 (46) real-space refinement was used to refine the model, imposing fivefold symmetry through NCS restraints detected from the reconstructed cryo-EM map (Correlation of symmetry-related regions: 0.96 and 0.97 for the two reconstructions). The model was manually adjusted in COOT 0.8.9.1 EL (49) and re-refined until the quality metrics were optimal and in agreement with the reconstruction. Model statistics are summarized in *SI Appendix*, Table S3. Additionally, the side chains and residues, where the density did not allow for confident building, were removed (*SI Appendix*, Table S4).

### Molecular Dynamics Simulations.

MD simulations were performed starting from the cryo-EM and X-ray structures resolved in the presence (PDB ID:s 7Q3G and 6V4S) and absence of calcium (PDB IDs: 7Q3H and 6V4A). For the X-ray structures, missing residues were built using MODELLER v. 9.22 (48); for the cryo-EM structures, the full-length models prior to removal of residues with insufficient density were used for simulation. Protonation states were set to neutral pH using PDB2PQR (50) or with CHARMM-GUI (51, 52), with equivalent result. The ions resolved in the with-calcium structures were retained for the simulations; additionally, a system was prepared from the with-calcium X-ray structure where the resolved ions were removed. For the X-ray structure resolved in the absence of calcium, three simulation systems were prepared: one without lipids intercalated between the subunits, one where an upper-leaflet 1-palmitoyl-2-oleoyl-sn-glycero-3-phosphocholine (POPC) lipid was manually placed guided by the intercalated head-group resolved in structure, and one with both an upper and a lower leaflet POPC lipid manually intercalated between subunits. Simulation systems were set up using the CHARMM-GUI membrane builder (51, 52), embedding the protein in a POPC bilayer, adding sodium and chloride ions to neutralize the system and to 150 mM NaCl concentration, and solvating in TIP3 water. Hydrogen mass repartitioning was used for the simulations starting from the cryo-EM structures. The CHARMM36m force-field was used (53), and simulations were performed using GROMACS-2020.3 (54). Systems were energy minimized, followed by equilibration for 2.25 ns during which restraints were gradually released. Four replicate production simulations of 1,000 ns were performed for each system.

Root mean square deviation (RMSD) and root mean square fluctuations (RMSF) were calculated using GROMACS. RMSDs were calculated for C-alpha atoms of full subunits and for individual structural domains, using the selection itself to align the trajectories and aligning on the ECD together with the TMD. RMSF was calculated for loop F, including the full residues and aligning on loop F. Distances and center of mass positions were tracked through the trajectories using VMD (55). Pore profiles for the simulations and for the structures were calculated using CHAP (56). PC analysis was performed using MDanalysis (57), first constructing a landscape using Cartesian coordinates from snapshots taken every 10 ns from all DeCLIC simulations. Snapshots and experimental structures were projected onto this landscape and colored by initial structure or by goodness of fit to SANS data. VMD (55) and UCSF Chimera (58) were used to render images.

## Supplementary Material

Appendix 01 (PDF)

Movie S1. In the absence of calcium, sodium (cyan and blue) visit the calcium binding site, and some sodium ions pass between the central ECD vestibule and the outside of the protein through this site (one instance highlighted in blue). Glutamates in one calcium binding site are shown as sticks, and for clarity only sodium ions in the vicinity of this site are shown. The movie covers the final 300 ns of one replica, and was generated with trajectory smoothing on.

## Data Availability

Cryo-EM density maps of the pentameric ligand-gated ion channel DeCLIC in detergent micelles have been deposited in the Electron Microscopy Data Bank under accession numbers EMD-13791 (pH 7, 10 mM Ca^2+^) and EMD-13792 (pH 7, no Ca^2+^). Each deposition includes the cryo-EM sharpened and unsharpened maps, both half-maps and the mask used for the final FSC calculation. Coordinates of the two models have been deposited in the Protein Data Bank. The accession numbers for the two DeCLIC structures are 7Q3G (pH 7, 10 mM Ca^2+^) and 7Q3H (pH 7, no Ca^2+^). The raw experimental SANS data are available from https://doi.ill.fr/10.5291/ILL-DATA.8-03-1002, and processed SANS data—with best-fitting models for the with-calcium dataset—are available in the Small Angle Scattering Biological Data Bank (SASBDB) as entries SASDNG5 and SASDNH5. MD simulation trajectories are available at Zenodo.org as entry https://doi.org/10.5281/zenodo.6022369.
