# Supplementary material for: Biophysical characterization of calcium-binding and modulatory-domain dynamics in a pentameric ligand-gated ion channel
Source: Proc Natl Acad Sci U S A. 2022 Dec 8;119(50):e2210669119. doi: 10.1073/pnas.2210669119 (PMC9897478; doi:10.1073/pnas.2210669119)
Supplement: Supplementary file 1 — Appendix 01 (PDF) [file pnas.2210669119.sapp.pdf]

Supporting Information for

Biophysical characterization of calcium-binding and modulatory-domain dynamics in a pentameric ligand-gated ion channel

Marie Lycksell, Urška Rovšnik, Anton Hanke, Anne Martel, Rebecca J Howard, and Erik Lindahl

Corresponding Erik Lindahl.
E-mail: erik.lindahl@scilifelab.se

This PDF file includes:

Figs. S1 to S19
Tables S1 to S7
Legend for Movie S1
SI References

Other supporting materials for this manuscript include the following:

Movie S1

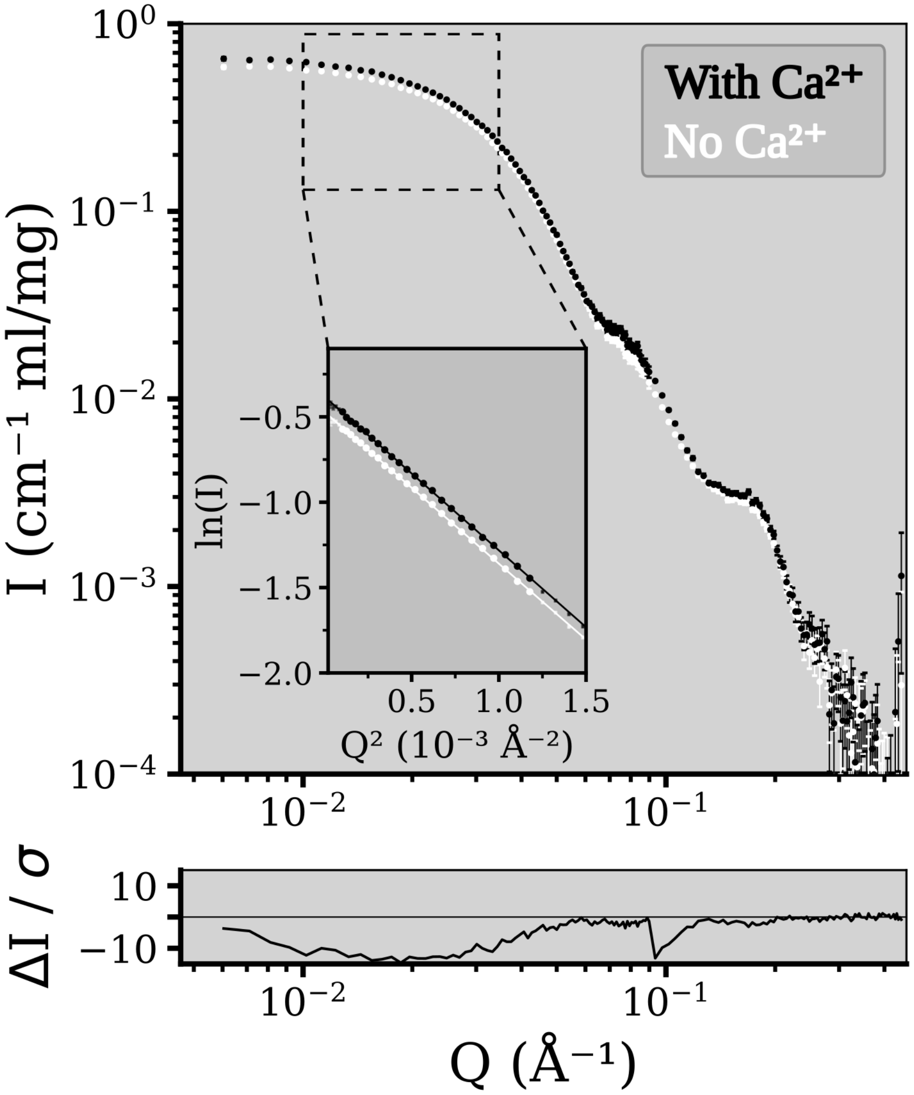

Fig. S1. Comparison of small angle neutron scattering from DeCLIC in the presence and absence of calcium. Small angle neutron scattering profiles from DeCLIC in the presence (black) and absence (white) of calcium, with the insert showing the Guinier plot of the low Q region together with the Guinier fits as lines. The lower panel shows the error weighted residual between the conditions.

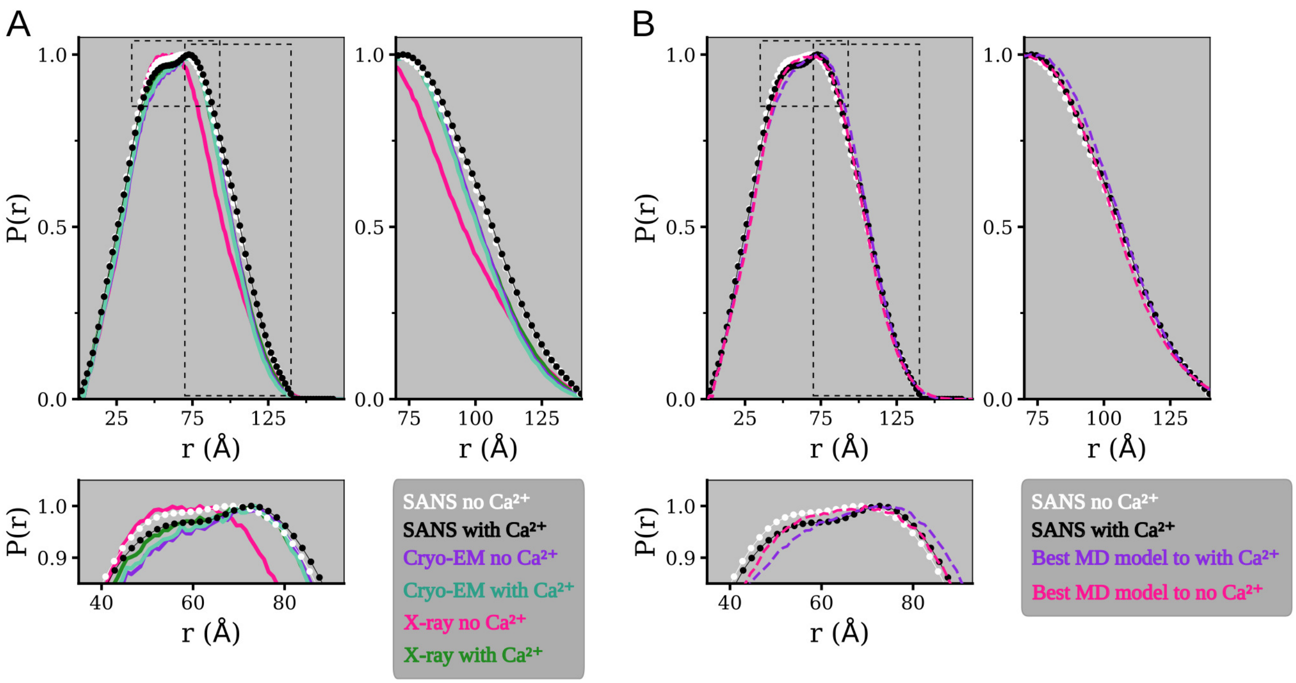

Fig. S2. Pairwise distance distributions from SANS compared to that of structures and models. (A) Pairwise distance distribution from SANS data with (black) and without (white) calcium, and for structures from cryo-EM and X-ray crystallography determined in the presence (aquamarine for cryo-EM, green for X-ray) and absence of calcium (purple for cryo-EM, pink for X-ray). The lower box shows a zoom on the peak of the distributions, the box to the right shows a zoom on the larger distances. The peak show that SANS in the absence of calcium yields a distribution with features similar to both the X-ray no Ca^{2+} structure and to the closed-like structures, while the SANS data with Ca^{2+} is closest to the X-ray with calcium structure. The long distances show that the structures in this region underestimate the values observed with SANS. (B) Pairwise distance distribution from SANS data with (black) and without (white) calcium, and for the models from molecular which gives the best fit to the SANS data. The purple model gives the best fit to the with Ca^{2+} SANS data and comes from the simulations of the calcium free cryo-EM structure. The pink model gives the best fit to the no Ca^{2+} SANS data and comes from the simulations of the calcium free X-ray structure. The lower box shows a zoom on the peak of the distributions, the box to the right shows a zoom on the larger distances. It can be seen in the peak region that these models are not perfect matches for the distance distributions obtained from SANS, while the larger distances show that the agreement between the models and the SANS data has improved in this region.

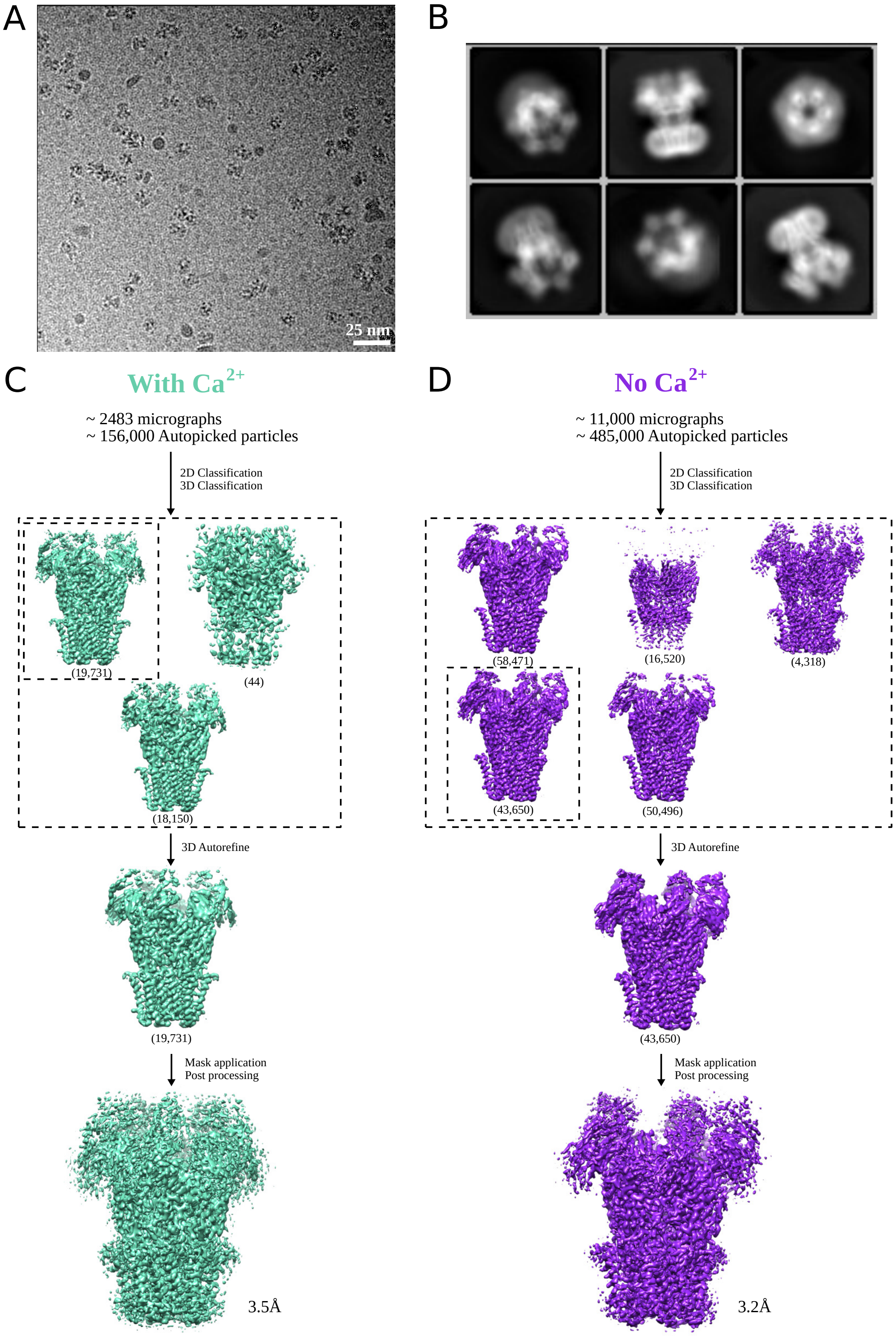

Fig. S3. Cryo-EM data and processing. (A) Representative micrograph from one of the grids used for data collection on a Titan Krios, showing detergent-solubilized DeCLIC particles. (B) Representative 2D class averages at 0.82 Å/px in a 256 x 256 pixel box and a 200 Å mask. (C) Overview of the cryo-EM processing pipeline for data collected at pH 7 with Ca^{2+}. (D) Same as in C but for a dataset where no Ca^{2+} was present (see Methods).

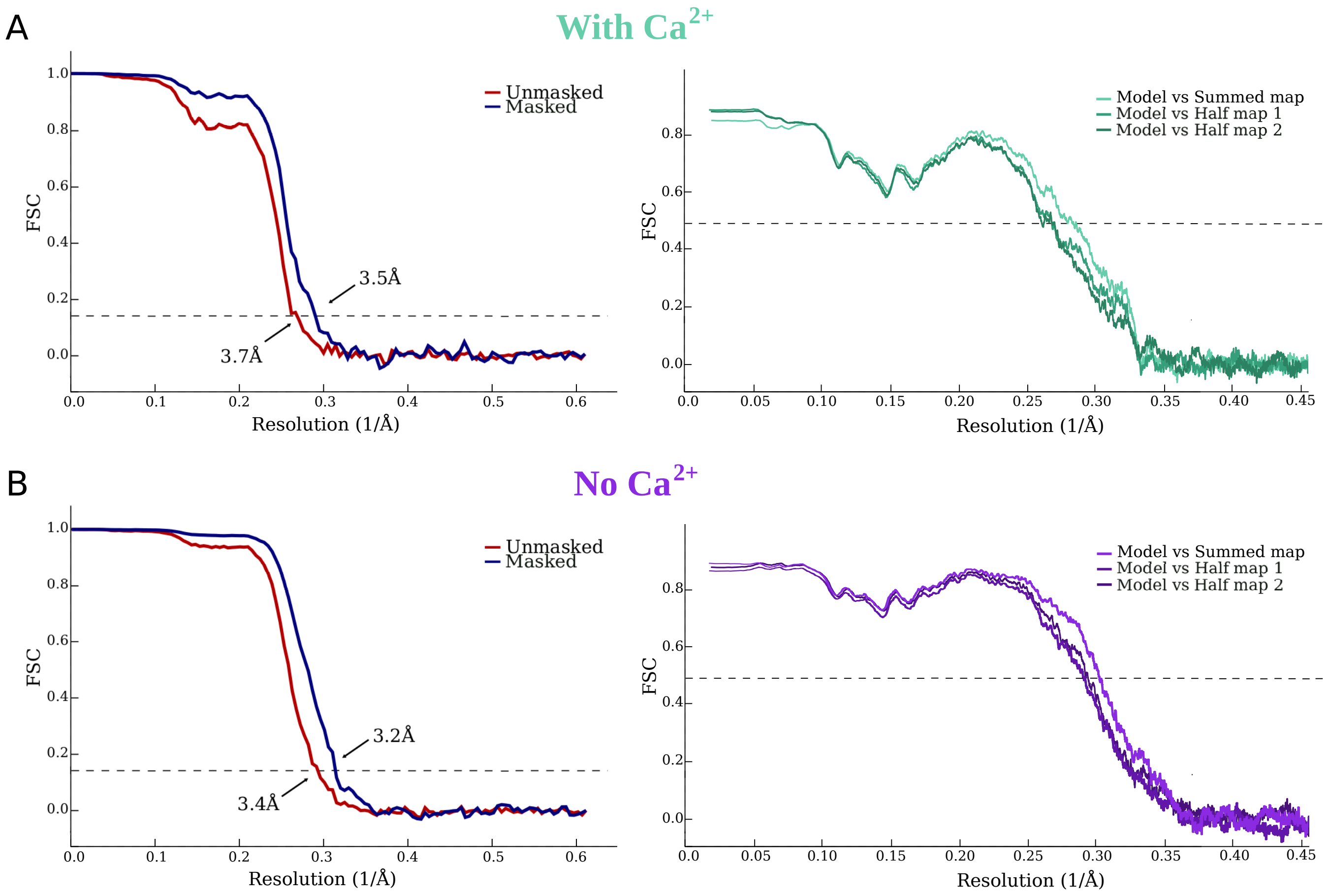

Fig. S4. Fourier Shell Correlation. (A) *Left*: FSC curves for cryo-EM reconstructions at pH 7 with Ca^{2+} before (red) and after (blue) applying a soft mask. *Right*: FSC curves for cross-validation between model and half-map 1 (medium), model and half-map 2 (dark) and model and summed map (light). (B) FSC curves represented as in panel A for the dataset at pH 7 without Ca^{2+}.

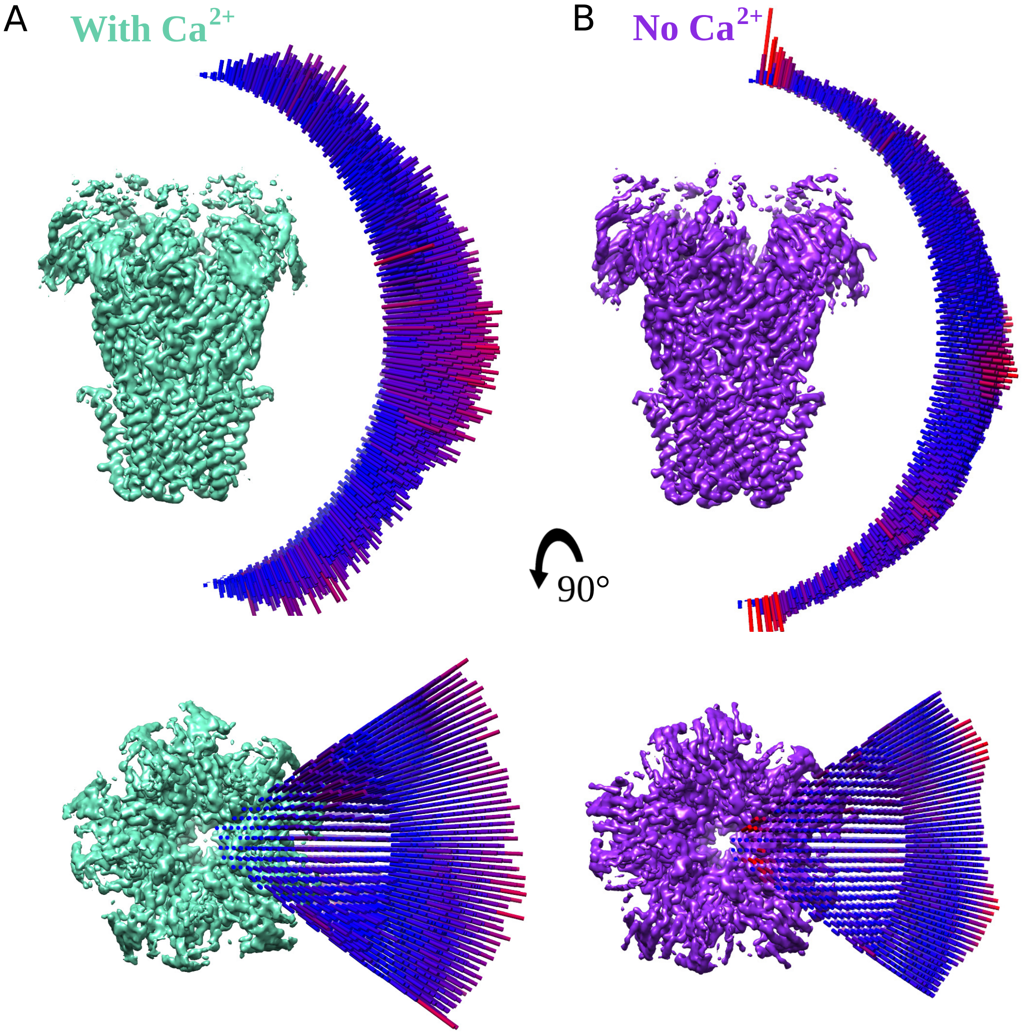

Fig. S5. Distribution of orientation sampling in the cryo-EM maps. (A) *Top*: Side view of the cryo-EM map for DeCLIC at pH 7 with Ca^{2+} and the distribution of orientation sampling after refinement. The height and the color (blue to red) of the bar represent the abundance of that particular view. *Bottom*: Representation of the same map from the top. (B) Map and distributions as in panel A for the dataset at pH 7 with no Ca^{2+}.

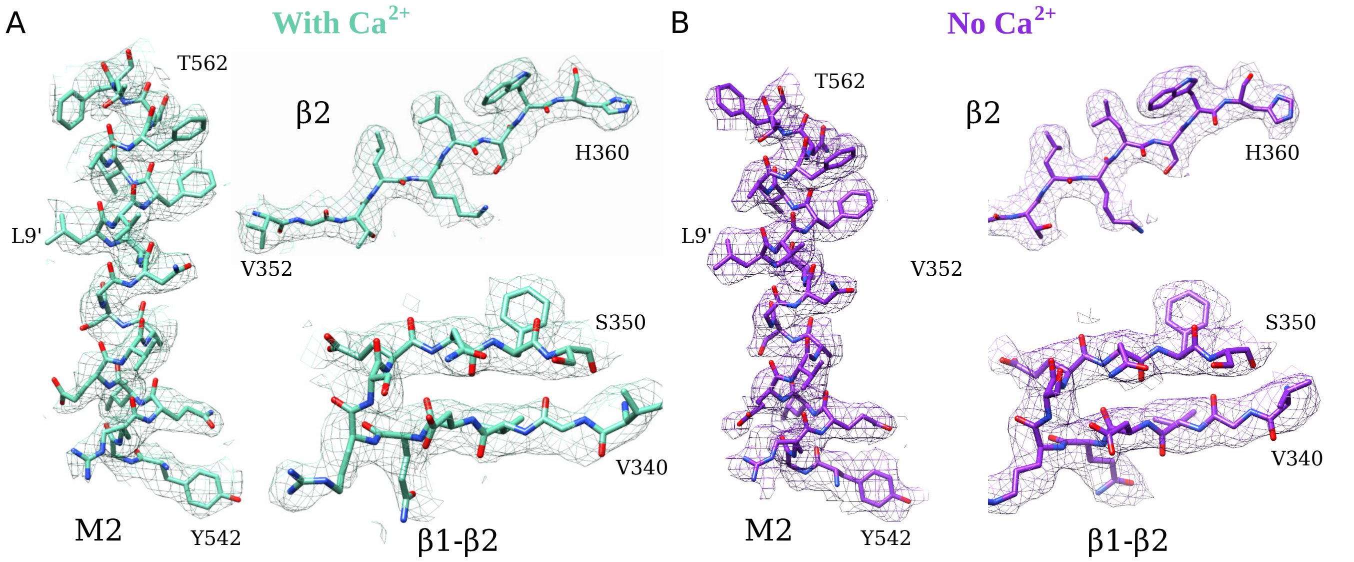

Fig. S6. Cryo-EM map and atomic model for select regions of DeCLIC. (A) Density (mesh) and corresponding atomic model (sticks, colored by heteroatom) for the M2 helix (Y542–T562), part of the β2 strand (V352-H360) and a β1-β2 loop (V340-S350) for the pH 7 with Ca^{2+} model. (B) Density and corresponding model, shown as in panel A, for the pH 7 model without Ca^{2+}.

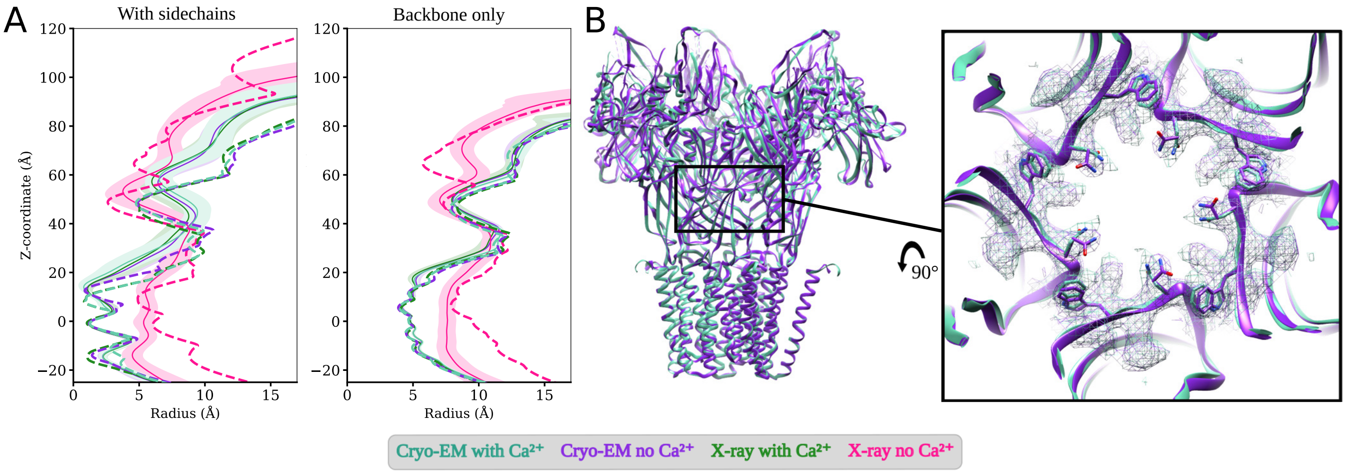

Fig. S7. Pore profiles of DeCLIC and zoom in view of the constriction point in the ECD. Pore profiles were calculated including sidechains (left) and only backbone atoms (right) respectively. Dashed lines show the profile for the starting structures and solid lines represent the simulation average with standard deviation in colored fill. The pore profiles for simulations of the with calcium X-ray structure and both cryo-EM structures overlap, and are in close agreement. The simulations started from the calcium free X-ray structure show a decrease in pore radius, largely attributable to backbone motion. Zoom in box (right) shows the most constricted region in the ECD with Trp407 and Asn405 being the two constricting residues.

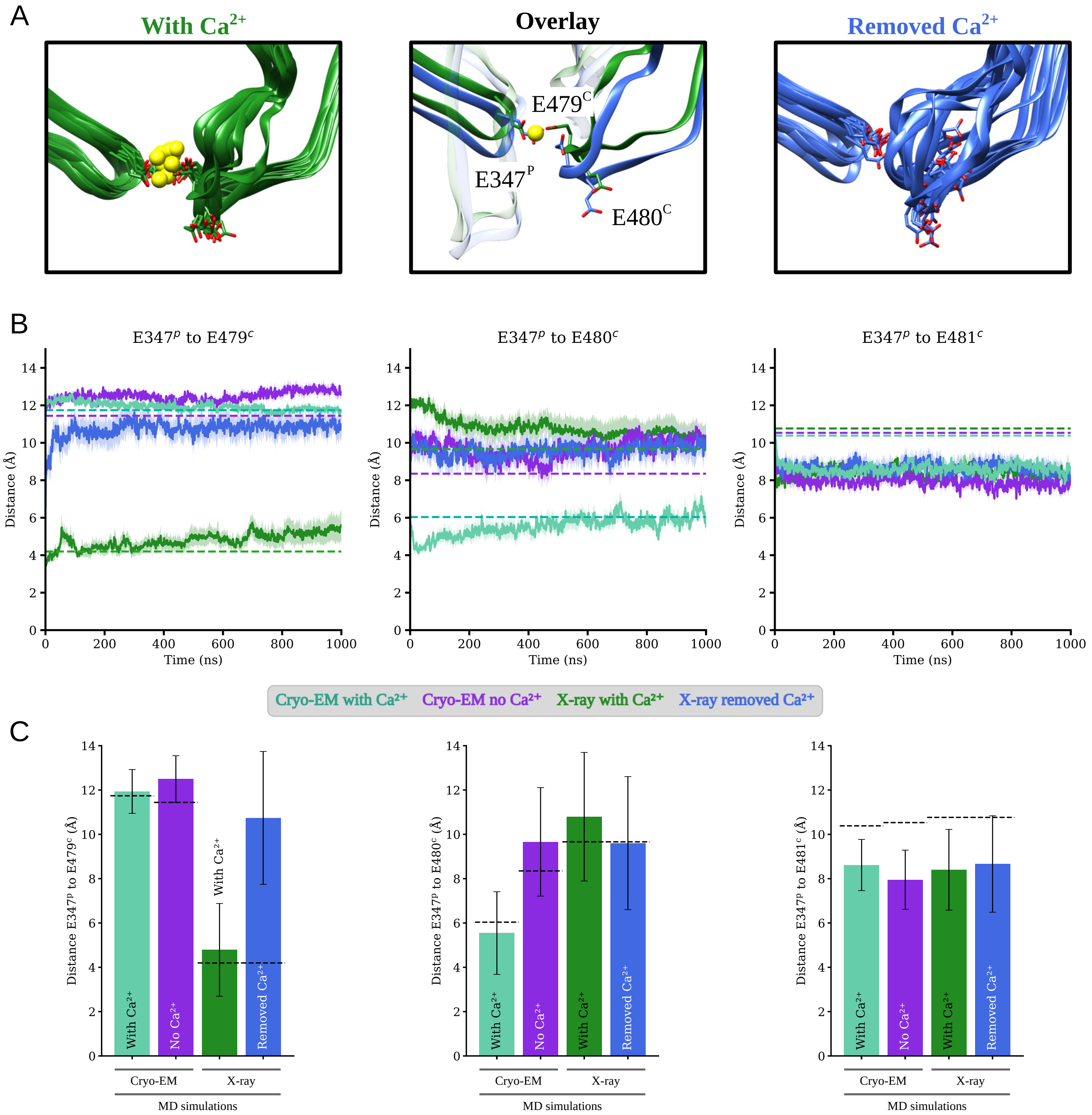

Fig. S8. Behaviour of glutamates in the calcium binding site. (A) Snapshots from simulations starting from the with calcium X-ray structure by Hu et al. (1) (PDB ID: 6V4S); in green simulated with the resolved calcium ion present, and in blue simulated with the calcium ion removed. The center panel shows an overlay the initial simulation frame, the left and right panels show simulation snapshots spaced by 100 ns. (B) Distance between the sidechain carboxyl groups of glutamates in the calcium binding site, tracked through the MD simulations. The average value from the simulations, averaged over all subunit interfaces from four replicas, are shown as solid lines with the standard error of the mean in colorfill. Dashed lines show the values measured in the X-ray and cryo-EM structures. (C) Distance between the sidechain carboxyl groups of glutamates in the calcium binding site, averaged over time, and all subunit interfaces from four replicas. Error bars show standard deviation. Dashed lines show the distance in the cryo-EM or X-ray structure from which the simulation was started.

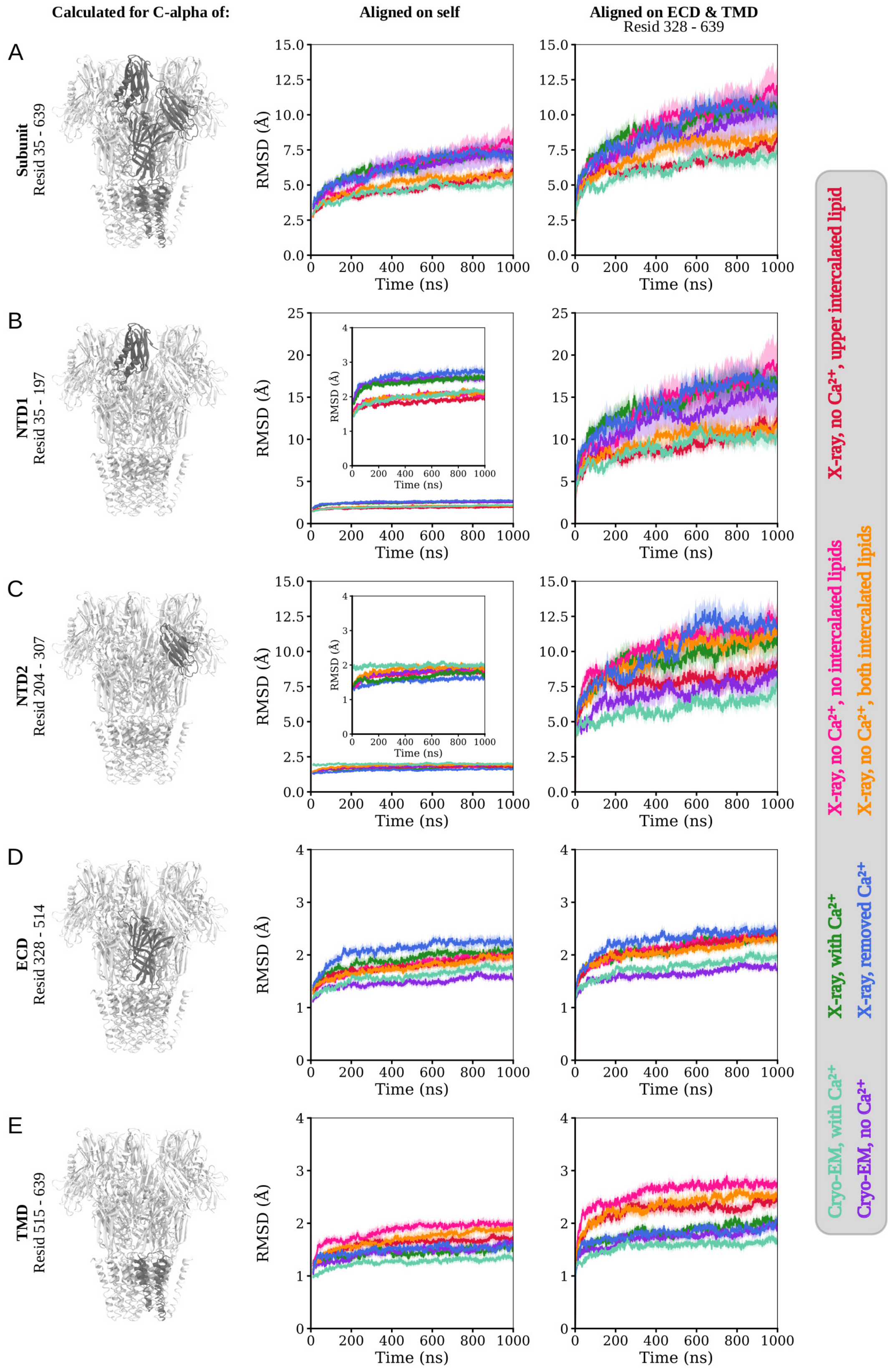

Fig. S9. Root mean square deviation (RMSD) for the simulations of DeCLIC. Solid lines are averages over all subunits in four replicates, the standard error of the mean is shown as color fill. The RMSD has been calculated for the full subunit (row A), and for the four different domains in DeCLIC (rows B through E), aligning on either the domain itself (center column), or aligning on the joint ECD and TMD of the chain (right column). Rows are shown with the same scale for the RMSD values, inserts are shown in cases where the alignments yielded very different RMSD values. For the NTD lobes the RMSD is notably higher when aligning on the ECD-TMD compared to when alignment is done on the domain itself, indicating that these domains undergo translation relative to the ECD-TMD part of the protein.

Marie Lycksell, Urška Rovšnik, Anton Hanke, Anne Martel, Rebecca J Howard, and Erik Lindahl

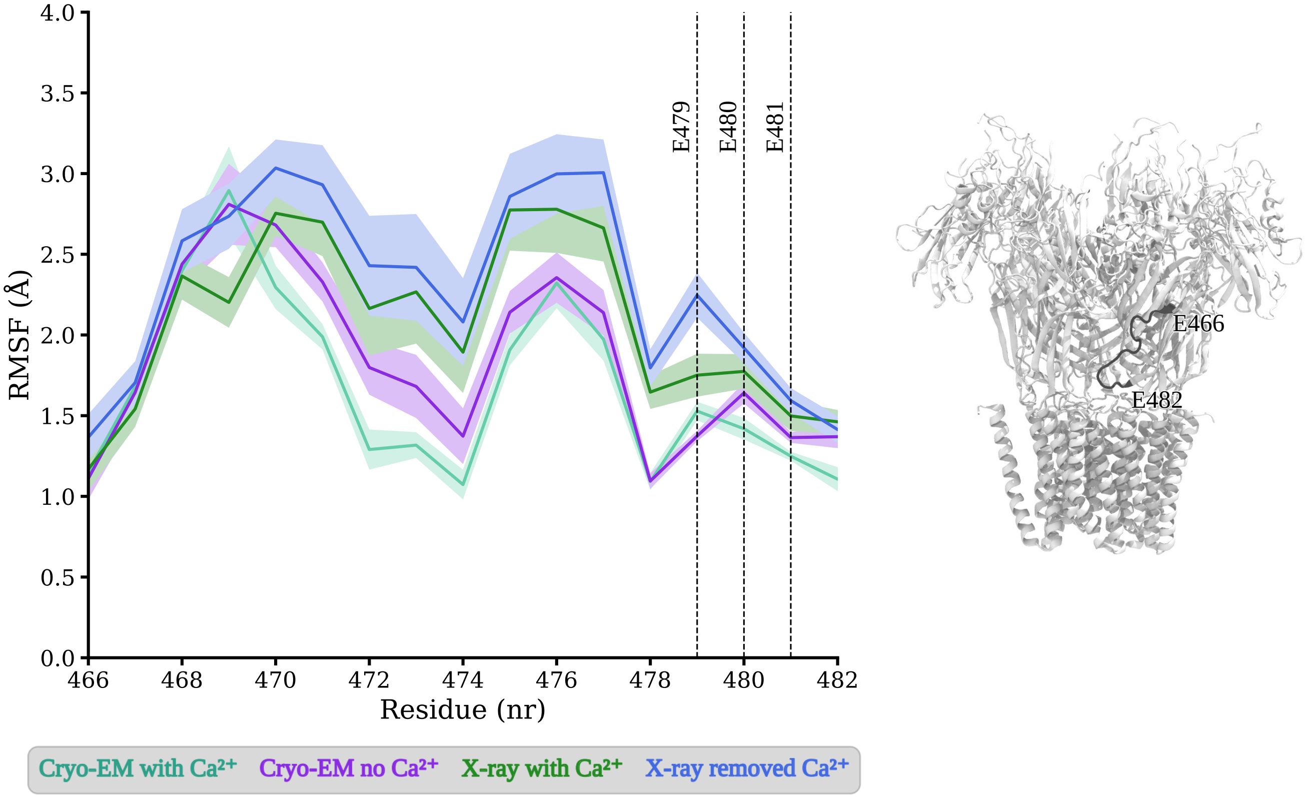

Fig. S10. Root mean square fluctuation (RMSF) for residues in loop F (residues shown in black on the structure shown to the right) from simulations starting from cryo-EM structures of DeCLIC in the presence (aquamarine) and absence (purple) of calcium, and from the X-ray structure with calcium - which was simulated with Ca^{2+} (green) and with Ca^{2+} removed (blue). Solid lines represent the average over all subunits in four replicas, and the standard error of the mean is shown as colorfill. Dashed vertical lines indicate the three consecutive glutamates E479, E480, and E481, which are located in the calcium binding site.

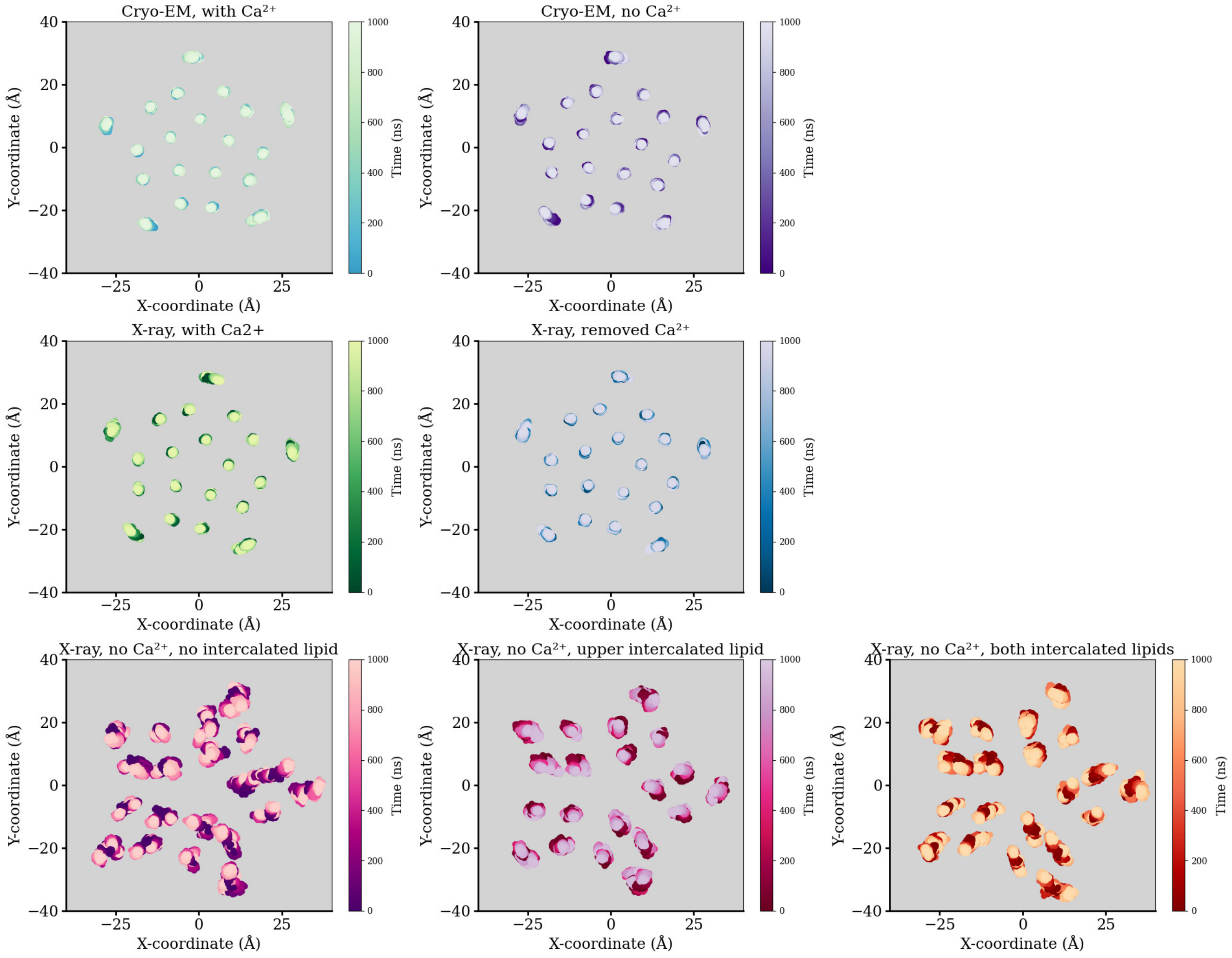

Fig. S11. Center of mass position for the transmembrane helices through molecular dynamics simulations, four replicas, of the cryo-EM structures with and without Ca^{2+}, the X-ray structure with Ca^{2+} (simulated with Ca^{2+} and with Ca^{2+} removed), and the X-ray structure without Ca^{2+} (simulated without intercalated lipid, with intercalated upper leaflet lipid, and with both upper and lower leaflet lipids intercalated). Simulations started from the cryo-EM structures and the with Ca^{2+} X-ray structure have stable helix positions through the simulations, with the most mobility for the lipid facing M4 helix. Simulations started from the no Ca^{2+} X-ray structure display less stable positions of the transmembrane helices.

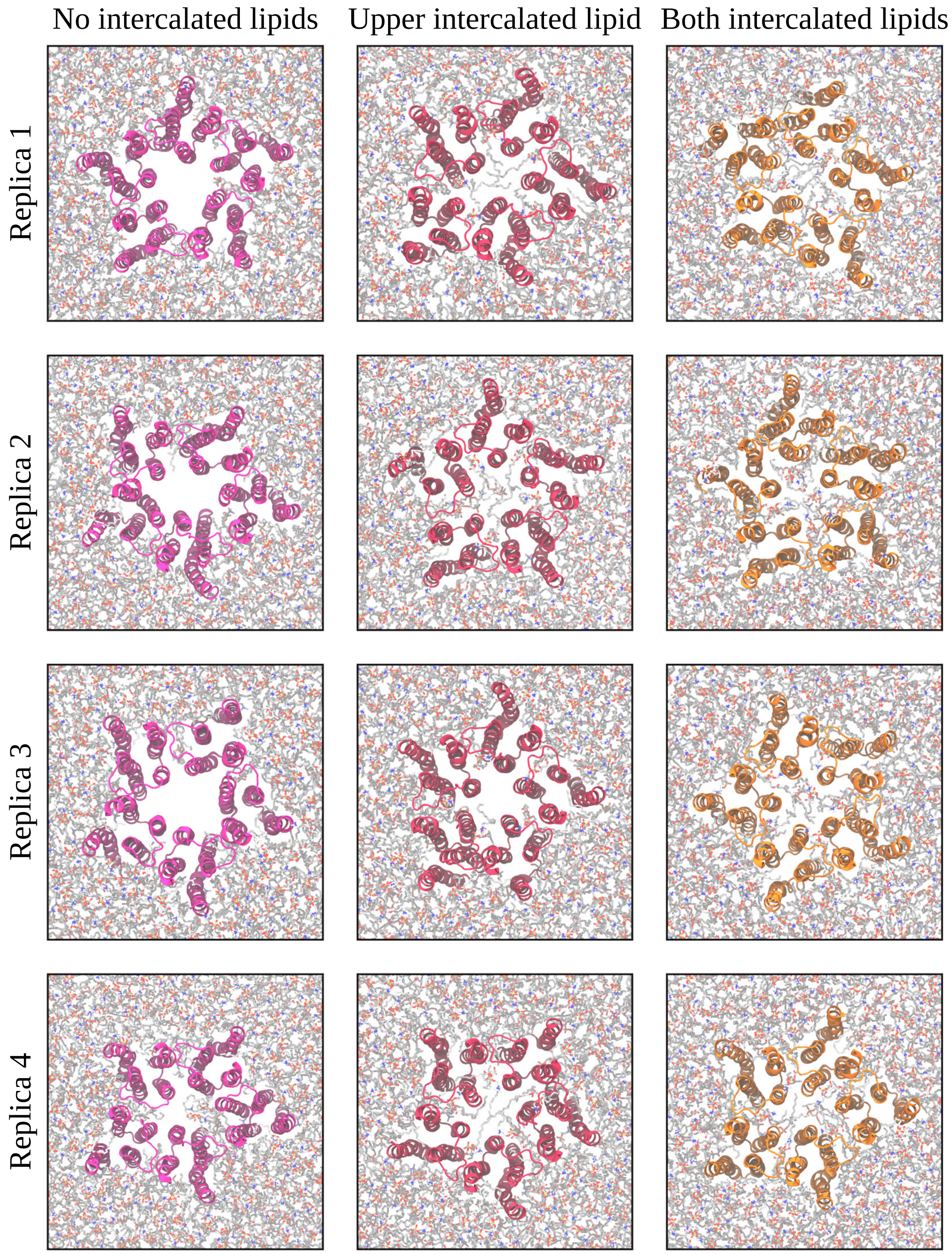

Fig. S12. Final frame (1000 ns) of the simulations started from the X-ray structure with no calcium, showing the transmembrane domain and lipid bilayer seen from the extracellular side. The left column show simulations started without a lipid intercalated between the subunits, the center column simulations where an upper leaflet lipid had been intercalated under the M2-M3 loop, and the right column show simulations with both an upper and a lower leaflet lipid intercalated. The pore lining M2 helices deviated during the simulations from their symmetric starting positions, and especially the systems started with intercalated lipids were prone to have lipids penetrate into the pore.

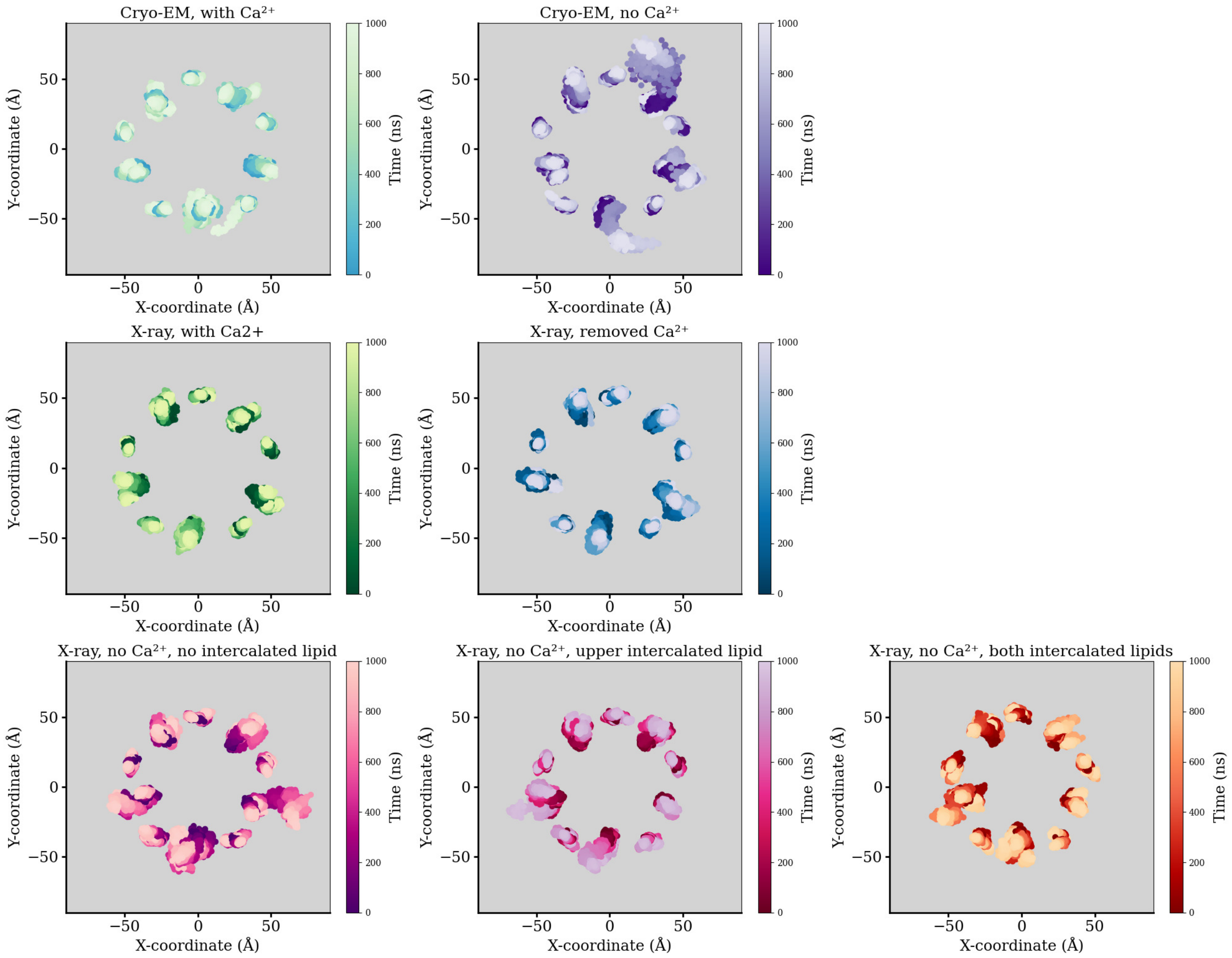

Fig. S13. Position of the center of mass of NTD1 and NTD2 through the MD simulations, shown on the XY-plane. The larger clusters are from NTD1, and the smaller ones are from NTD2. Values from four replicate simulations are plotted for each system.

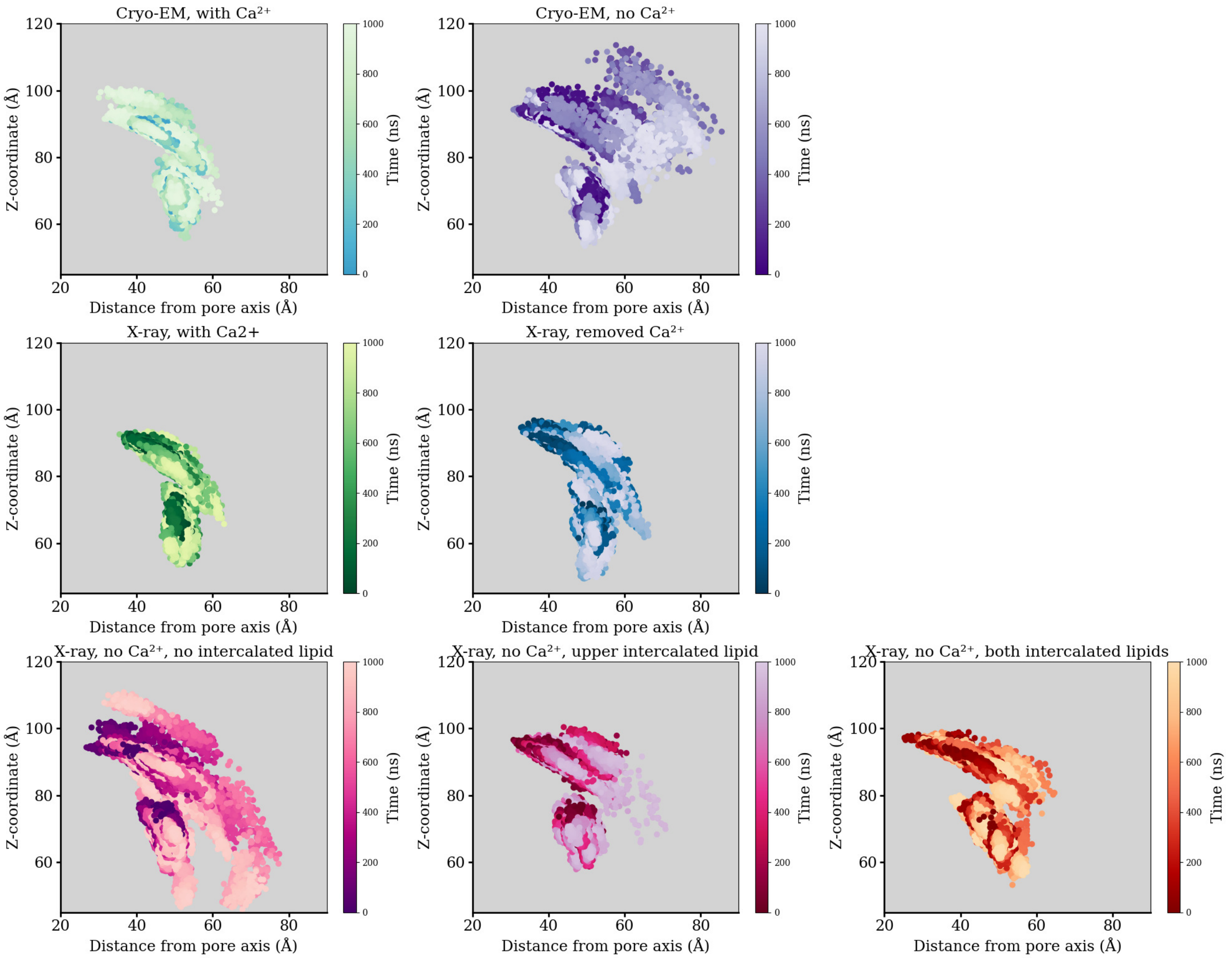

Fig. S14. Position of the center of mass of NTD1 and NTD2 through the MD simulations, shown as Z-coordinate versus distance from pore axis. The upper cluster is from NTD1:s, and the lower from NTD2:s. Values from four replicate simulations are plotted for each system, and NTD lobes from separate subunits are individually represented.

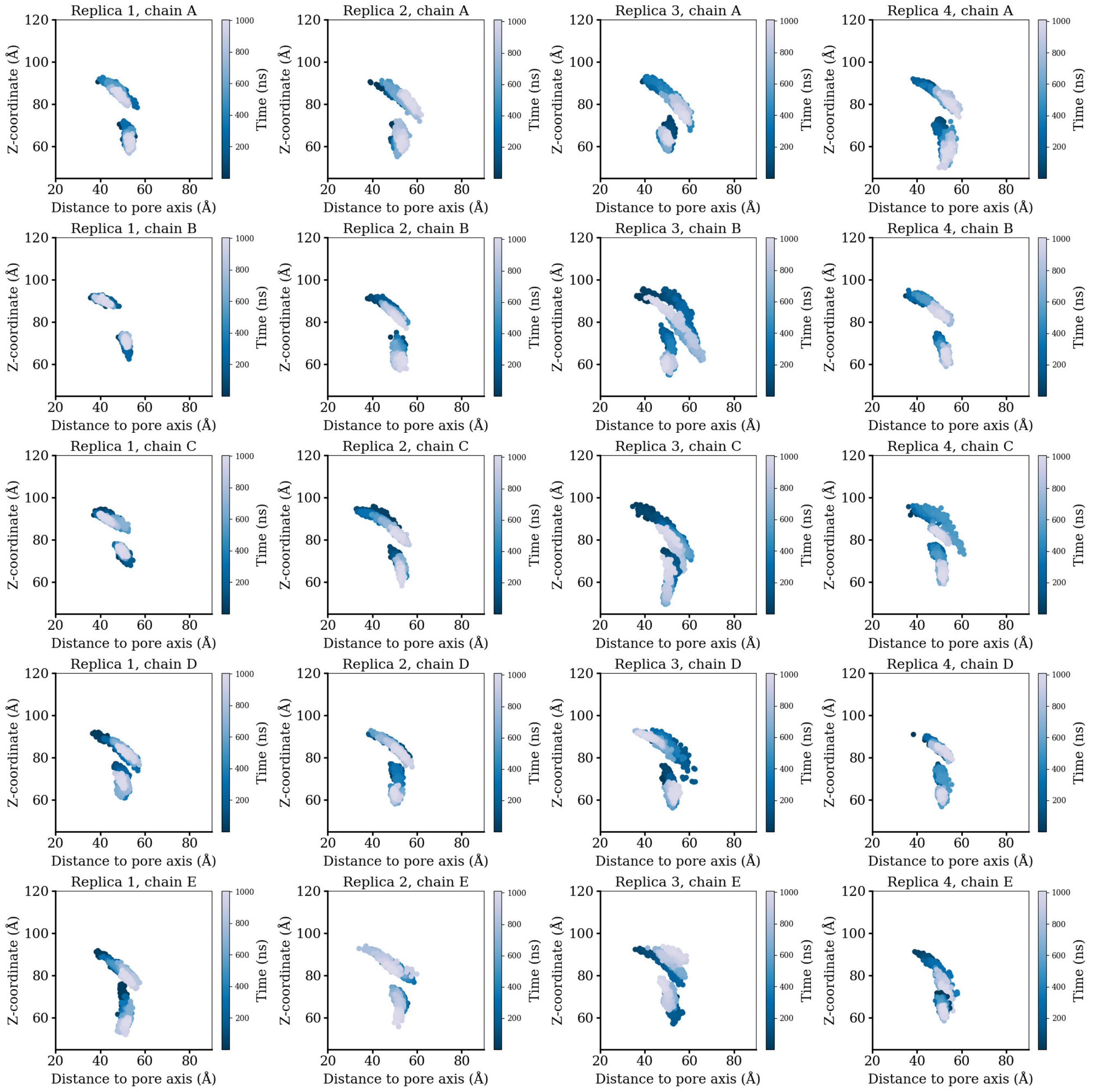

Fig. S15. Center of mass position of NTD1 and NTD2 shown for individual subunits across four simulation replicas of the closed-like X-ray structure with Ca^{2+} removed for the simulations. The upper cluster is from NTD1, and the lower from NTD2. The simulations contain examples of the domains remaining close to the starting positions (e.g. replica 1 chain B: column 1, row 2), of NTD1 moving out-and-down and staying there (e.g. replica 2 chain C: column 2, row 3), and of NTD1 visiting the out-and-down conformation before returning to the starting position (e.g. replica 3 chain B: column 3, row 2).

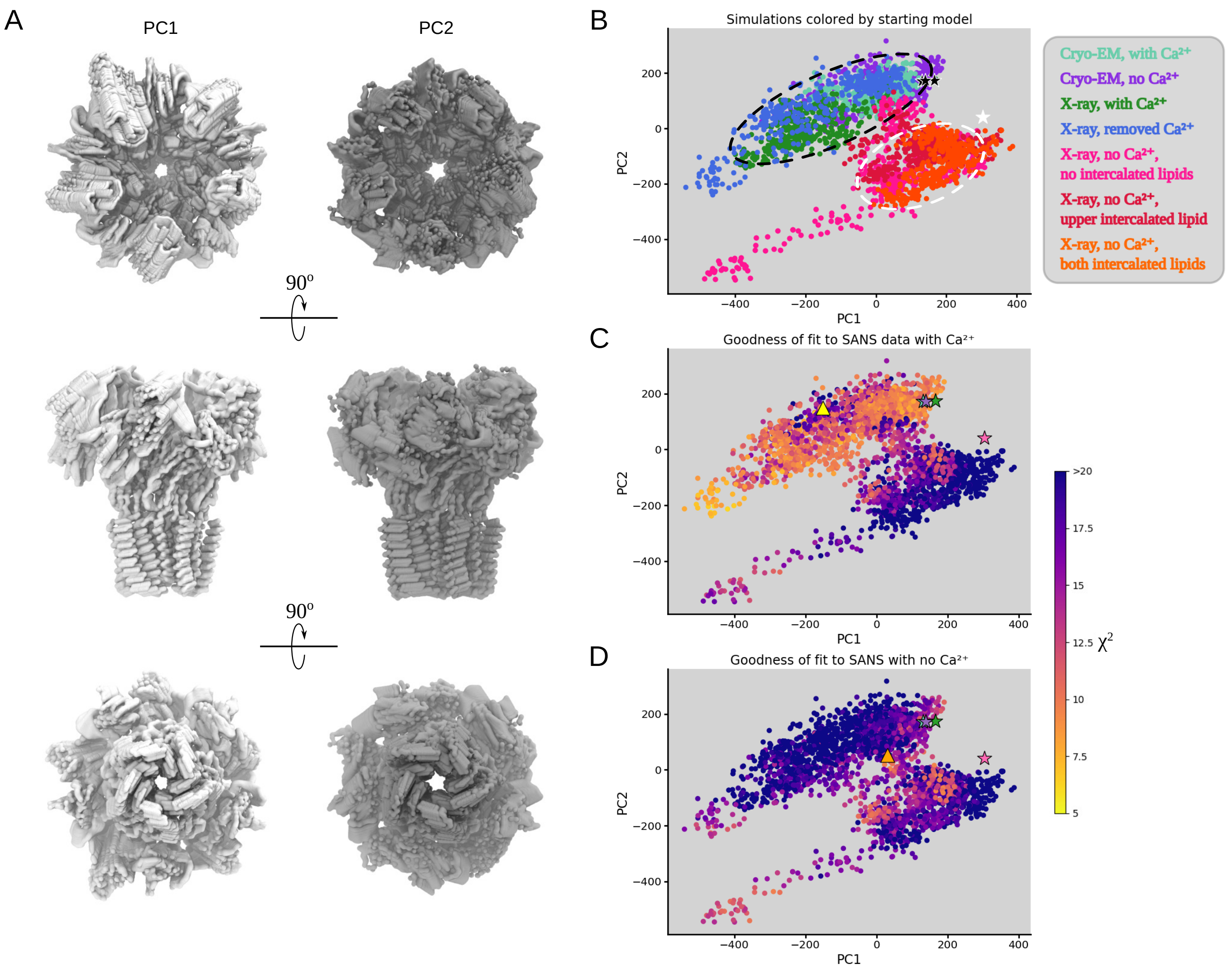

Fig. S16.

textbfPrincipal component analysis of DeCLIC simulations (A) Representations of the motions described by the first (PC1) and second (PC2) principal components based on analysis of all DeCLIC simulations in Cartesian space, viewed from the periplasmic side (top), the membrane plane (middle), or the intracellular side (bottom). (B) MD-simulation snapshots, taken every 10 ns and colored by system, projected on their first two PCs. Closed-state simulations were initiated from structures determined by cryo-EM in the presence (teal) or absence (purple) of calcium in this work, or by crystallography with calcium retained (green) or removed (blue) from PDB ID 6V4S. Open-state simulations were initiated from the structures determined by crystallography as reported in PDB ID 6V4A (pink), or with lipids intercalated in the outer (red) or outer and inner (orange) membrane leaflets. Black and white stars represent projections of experimental structures with closed and open pores, respectively. Black and white circles indicate closed- and open-state clusters, respectively. (C) Snapshots as in A colored by goodness of fit to SANS data collected in the presence of Ca2+, according to the scalebar to the right. The snapshot with the best fit is indicated by a triangle in equivalent coloring. Stars represent projections of experimental structures, colored by system as in A. (D) Snapshots as in B colored by goodness of fit to SANS data in the absence of Ca2+.

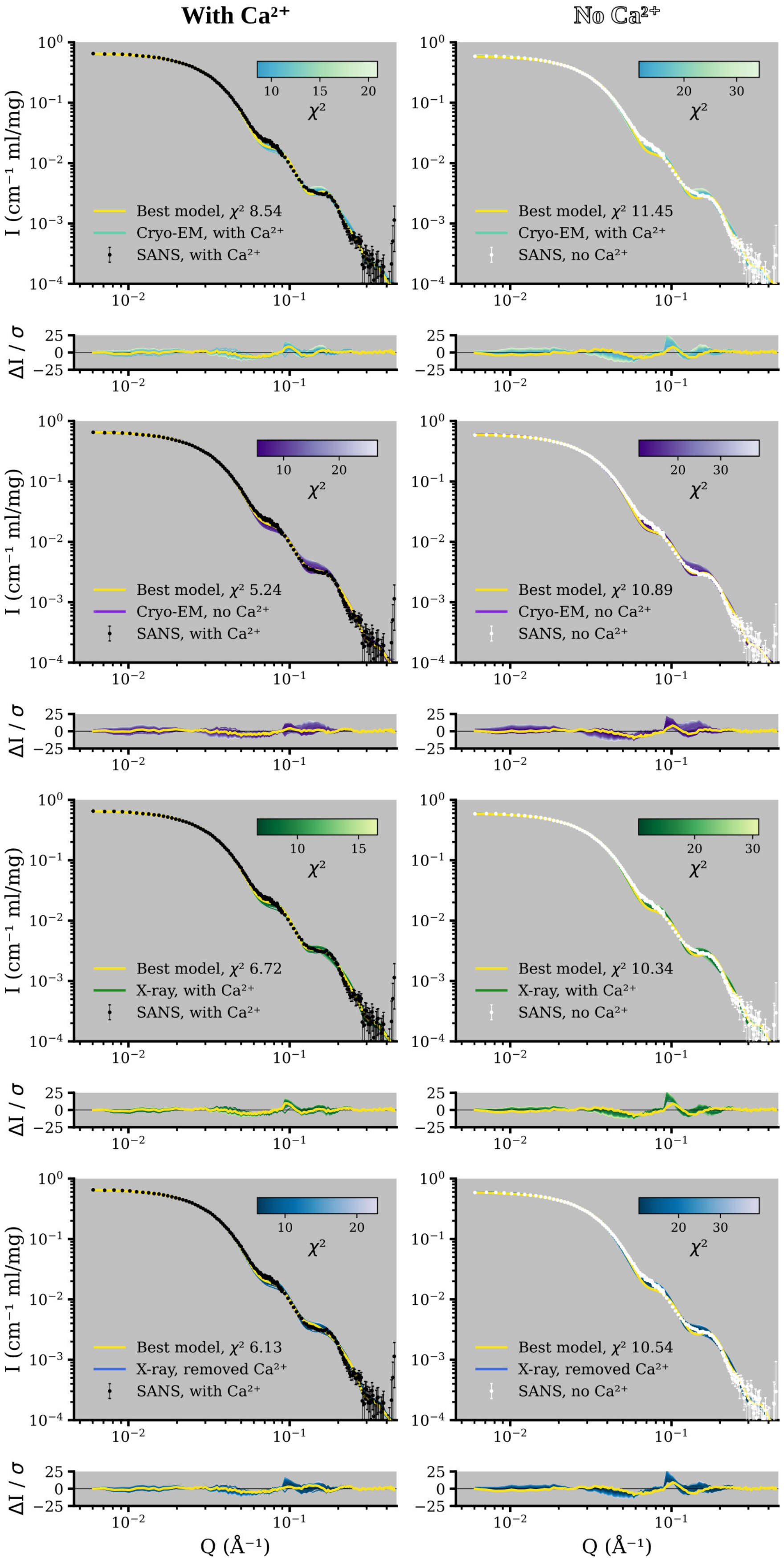

Fig. S17. Fits to the SANS data collected with (black) and without (white) calcium for models from simulations started from closed-like structures, i.e. the cryoEM structures and the with calcium X-ray structure. For each simulation system the theoretical curve from the snapshot yielding the best goodness of fit to the SANS data is shown in yellow.

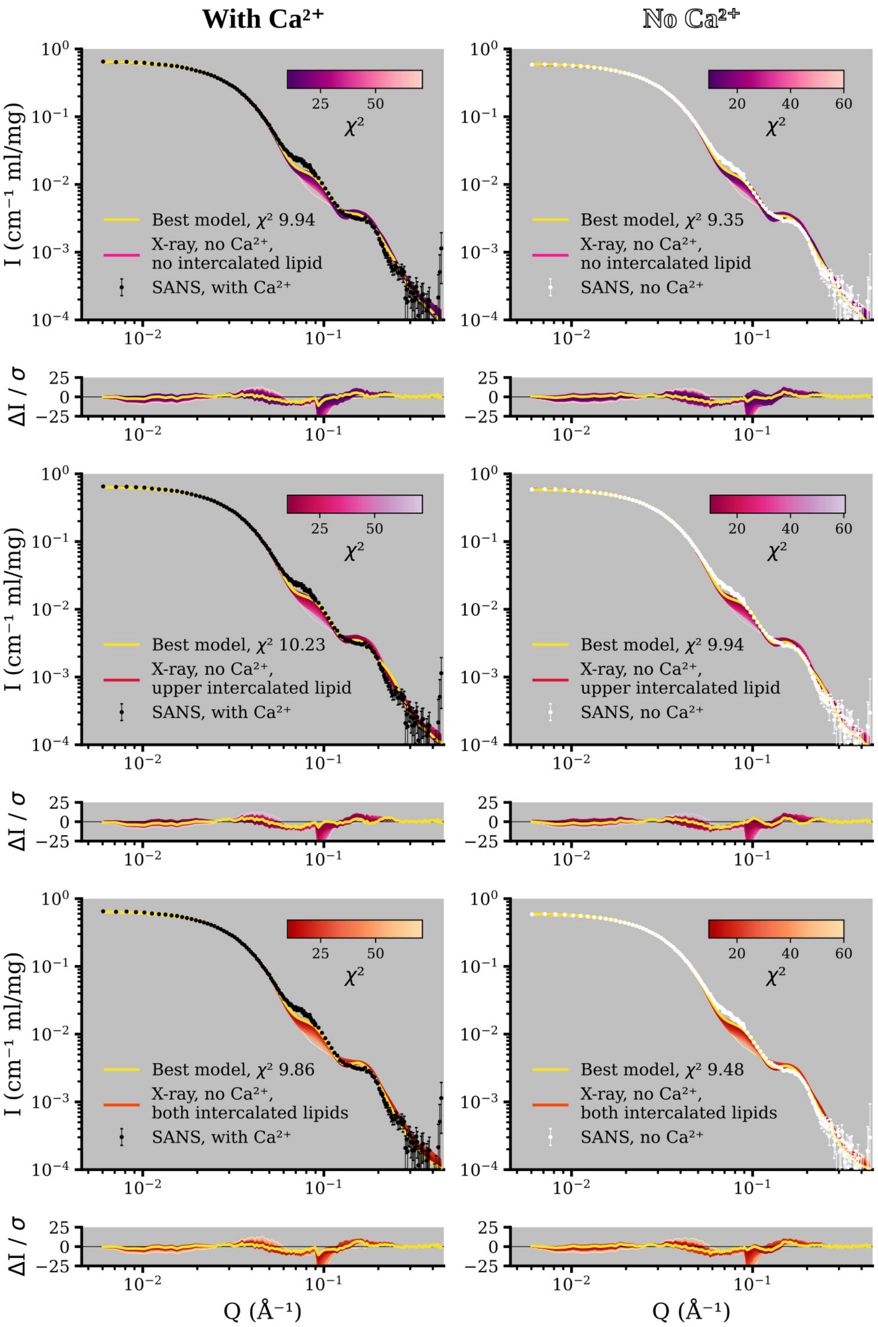

Fig. S18. Fits to the SANS data collected with (black) and without (white) calcium for models from simulations started from the no calcium X-ray structure. For each simulation system the theoretical curve from the snapshot yielding the best goodness of fit to the SANS data is shown in yellow.

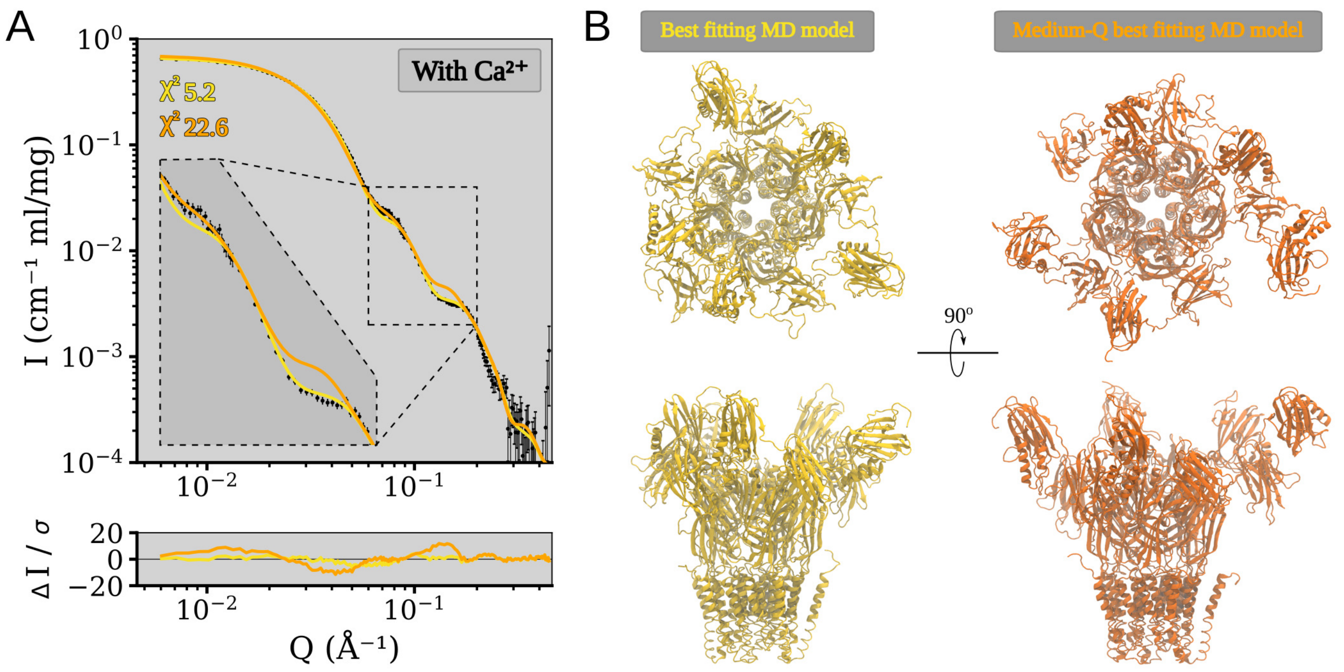

Fig. S19. Comparison of the model with best overall fit to the with calcium SANS data and the model with the best fit to the $Q \in [0.06, 0.09]$ Å^{-1} feature. (A) Theoretical curves from MD simulation snapshots of the cryoEM no Ca^{2+} structure, fitted to the with Ca^{2+} SANS data. In yellow is the snapshot which yielded the best over all fit to the SANS data (χ^2 of 5.2), and in orange the one which had the best agreement with the medium-Q feature ($Q \in [0.06, 0.09]$ Å^{-1}, χ^2 to full scattering profile of 22.6). The model with the best agreement with the medium Q-feature has worse fit at both higher and lower Q, as seen in the zoomed insert, and in the error weighted residual. (B) Top and side view of the the snapshot yielding the fits in panel A. Both models are asymmetric, and have different degrees of extension in the NTD.

Table S1. Summary of structural parameters calculated from the SANS data. For the Guinier analysis the minimum and maximum Q-value included is also listed.

	With Ca^{2+}	No Ca^{2+}
Guinier analysis		
$I(0)$ (cm^{-1})	0.68	0.62
R_g (Å)	52.0 $\pm$ 0.2	51.6 $\pm$ 0.2
Q_{min} (Å^{-1})	0.010	0.010
Q_{max} (Å^{-1}), (QR_g max)	0.035 (1.7)	0.035 (1.7)
Coefficient of correlation R^2	0.9997	0.9997
M from $I(0)$ (kDa), (ratio to predicted)	348 (1.02)	318 (0.93)
$P(r)$ analysis		
$I(0)$ (cm^{-1})	0.679	0.621
R_g (Å)	51.26 $\pm$ 0.03	50.90 $\pm$ 0.03
d_{max} (Å)	139.77 $\pm$ 0.74	139.26 $\pm$ 0.56
q range (Å^{-1})	0.0060 - 0.4453	0.0060 - 0.4452
χ^2	0.91	0.71
PepsiSANS		
R_g (Å)	50.2	49.3

Table S2. Summary of modeling using protein structures and models based on protein structures, covering the radius of gyration of the model and the χ^2 goodness of fit to the experimental data for the model.

Structures	6V4A	6V4S	Cryo-EM No Ca^{2+}	Cryo-EM With Ca^{2+}	
PepsiSANS					
Predicted R_g (Å)	48.2	49.5	49.6	49.2	
SANS with Ca^{2+} χ^2	79.23	10.75	11.18	12.12	
SANS no Ca^{2+} χ^2	67.70	8.77	11.71	11.26	
MD-simulation models	6V4A No Ca^{2+}	6V4S With Ca^{2+}	6V4S Removed Ca^{2+}	Cryo-EM No Ca^{2+}	Cryo-EM With Ca^{2+}
Equilibration time (ns)	2.25	2.25	2.25	2.25	2.25
Simulation time (ns/replica)	1000	1000	1000	1000	1000
Replicas (nr)	4	4	4	4	4
χ^2 range (PepsiSANS)					
SANS with Ca^{2+}	9.94 - 71.33	6.72 - 16.54	6.13 - 22.86	5.24 - 26.92	8.54 - 20.93
SANS no Ca^{2+}	9.35 - 60.02	10.34 - 31.04	10.54 - 39.29	10.89 - 39.02	11.45 - 34.30

Table S3. Cryo-EM data collection and model refinement statistics

Data Collection	**With Ca^{2+}**	**No Ca^{2+}**
Microscope	FEI Titan Krios	
Magnification	165,000	
Voltage (kV)	300	
Electron exposure (e^-/Å^2)	~ 40	
Defocus Range (µm)	-2.0 – -3.6	-1.4 – -3.2
Pixel Size (Å)	0.82	
Symmetry Imposed	C5	
Number of Images	~ 2483	$\sim 11,505$
Particles Picked	$\sim 156,000$	$\sim 485,000$
Particles Refined	19,731	43,650
Refinement		
Resolution (Å)	3.5	3.2
FSC Threshold	0.143	
Map sharpening B-factor	-123	-100
Model Composition		
Non-hydrogen Protein atoms	19,570	21,620
Protein Residues	2585	2795
Ligands	5	0
B-factor (Å$^{2)}$	33.4	67.5
RMSD		
Bond Lengths (Å)	0.004	0.004
Bond Angles ($^\circ$)	0.65	0.58
Validation		
Molprobity Score	1.97	1.86
Clashscore	10.37	9.02
Poor Rotamers (%)	0	0
Ramachandran Plot		
Favored (%)	93.4	94.4
Allowed (%)	6.6	5.6
Outliers (%)	0	0

Table S4. Table of missing residues and sidechains from the cryo-EM reconstructions

	With Ca^{2+}	No Ca^{2+}
Removed Residues		
NTD1	28 – 68	28 – 37
		57 –67
	78 – 83	79 – 84
	101 – 104	
	118 – 120	
	141 – 148	142 – 145
	170 – 196	172 – 193
NTD2	237 – 245	
C-terminus	641 – 642	640 – 642
Total Removed	**99**	**56**
Removed Sidechains		
Leu	75, 96, 100, 110, 197, 208, 257, 261, 271, 303	110, 197, 208, 235, 261, 311
Ile	86, 107, 117, 137, 151, 199, 279	137, 199
Asp	87, 105, 115, 122, 215, 263, 278, 290, 294, 343, 361, 418, 435, 437	76, 115, 140, 213, 276, 361
Glu	97, 134, 209, 270, 297, 313, 425	46, 241, 270, 313
Arg	116, 211, 224, 301, 312	211
Lys	127	98, 127, 158, 214
Ser	132	132
Phe	135, 259	259
Asn	153, 310	104
Gln	277, 316	316
Val	319, 323	319
Thr		71

Table S5. Sample details.

Organism of origin	*Desulfofustis* sp. PB-SRB1
Expression system	*Echerichia coli*
UniProt ID	V4JF97
Extinction coefficient [A_{280} 0.1%(w/v)]	1.107
Volume from structure (Å^3)	495062
Particle contrast from sequence and solvent constituents, $\Delta\bar{\rho}$ ($\|\rho_{protein} - \rho_{solvent}\|$; 10^{10} cm^{-2})	3.94 ($\|2.44 - 6.38\|$)
M from chemical composition (kDa)	341.7

Table S6. Summary the SANS collection parameters, including wavelength, pathlength, detector distances, Q-range, absolute scaling method, normalization, sample concentration and volume, and buffer composition.

Instrument	ILL D22
Wavelength (Å)	6
Pathlength (cm)	0.1
Detector distances (m)	2.8m/2.8m & 8m/8m
Q measurement range (Å^{-1})	0.006 - 0.452
Absolute scaling method	Incident beam flux
Normalization	Divided by concentration
Exposure time (aggregate time)	
With Ca^{2+}	111 x 30s (55.5 min)
No Ca^{2+}	127 x 30s (63.5 min)
Column	Superdex 200 Increase 10/300
Sample temperature (^{o}C)	10
Loading concentration	6 mg/ml
Injection volume	240 µl
Flow rate (ml/min)	0.2, 0.01, 0
Average concentration (mg/ml) in combined data frames	
With Ca^{2+}	1.09
No Ca^{2+}	1.14
Solvent	
With Ca^{2+}	D_2O, 150 mM NaCl, 20 mM Tris·HCl, 10 mM $CaCl_2$, 0.5 mM d-DDM
No Ca^{2+}	D_2O, 150 mM NaCl, 20 mM Tris·HCl, 10 mM EDTA, 0.5 mM d-DDM

Table S7. Summary of software and equations employed for SANS data reduction, analysis, and interpretation.

Momentum transfer	Q=(4π/λ)sin(θ)
SANS data reduction	GRASP v. 9.04 (2)
Extinction coefficient estimate	ProtParam (3)
Guinier equation	$\ln(I(Q)) = \ln(I(0)) - \frac{R_g^2}{3}Q^2$
Calculation of M_w	$M_w = \frac{N_A \cdot I(0)}{c(\Delta\rho \cdot \bar{\nu})^2}$
Calculation of ρ	$\rho = (\sum_{i=1}^{N} b_i)/V$
Calculation of $\bar{\nu}$	$\bar{\nu} = V/M_{aa}$
Protein volume estimation	^{3}V: Voss Volume Voxelator (4)
Relationship between Q and distance	$d = 2\pi/$Q
$P(r)$ analysis	BayesApp (5) *via* web server (https://somo.chem.utk.edu/bayesapp/)
$P(r)$ from structure	CaPP (6) (https://github.com/Niels-Bohr-Institute-XNS-StructBiophys/CaPP)
Atomic structure modelling	PepsiSANS v. 3.0 (7)
Missing sequence modelling	MODELLER v. 9.22 (8)
Simulation system set-up	CHARMM-GUI membrame builder (9, 10)
Molecular dynamics simulations	GROMACS v. 2020.3 (11)
Theoretical R_g	PepsiSANS v. 3.0 (7)
Hydrogen-deuterium exchange	PSX (12)
Three-dimensional graphic model representation	VMD (13), UCSF Chimera (14)
Plots	MATPLOTLIB (15)
Pore profiles	CHAP (16)

Movie S1. In the absence of calcium, sodium (cyan and blue) visit the calcium binding site, and some sodium ions pass between the central ECD vestibule and the outside of the protein through this site (one instance highlighted in blue). Glutamates in one calcium binding site are shown as sticks, and for clarity only sodium ions in the vicinity of this site are shown. The movie covers the final 300 ns of one replica, and was generated with trajectory smoothing on.

References

1. H Hu, RJ Howard, U Bastolla, E Lindahl, M Delarue, Structural basis for allosteric transitions of a multidomain pentameric ligand-gated ion channel. *Proc. Natl. Acad. Sci.* **117**, 13437–13446 (2020).
2. C Dewhurst, *GRASP v. 9.04.* (Institute Laue-Langevin), (2020-08-24).
3. E Gasteiger, et al., Protein identification and analysis tools on the expasy server in *The proteomics protocols handbook.* (Springer), pp. 571–607 (2005).
4. NR Voss, M Gerstein, 3v: cavity, channel and cleft volume calculator and extractor. *Nucleic acids research* **38**, W555–W562 (2010).
5. S Hansen, Bayesapp: a web site for indirect transformation of small-angle scattering data. *J. Appl. Crystallogr.* **45**, 566–567 (2012).
6. AH Larsen, Capp: Calculating pair distance distribution functions for proteins (2020).
7. S Grudinin, *Pepsi-SANS v. 3.0.* (Nano-D team, Inria/CNRS Grenoble), (2020).
8. B Webb, A Sali, Comparative protein structure modeling using modeller. *Curr. Protoc. Bioinforma.* **54**, 5.6.1–5.6.37 (2016).
9. S Jo, T Kim, W Im, Automated builder and database of protein/membrane complexes for molecular dynamics simulations. *PloS one* **2**, e880 (2007).
10. S Jo, T Kim, VG Iyer, W Im, Charmm-gui: a web-based graphical user interface for charmm. *J. computational chemistry* **29**, 1859–1865 (2008).
11. MJ Abraham, et al., Gromacs: High performance molecular simulations through multi-level parallelism from laptops to supercomputers. *SoftwareX* **1**, 19–25 (2015).
12. MC Pedersen, et al., Psx, protein–solvent exchange: software for calculation of deuterium-exchange effects in small-angle neutron scattering measurements from protein coordinates. *J. Appl. Crystallogr.* **52**, 1427–1436 (2019).
13. W Humphrey, A Dalke, K Schulten, VMD – Visual Molecular Dynamics. *J. Mol. Graph.* **14**, 33–38 (1996).
14. EF Pettersen, et al., Ucsf chimera—a visualization system for exploratory research and analysis. *J. computational chemistry* **25**, 1605–1612 (2004).
15. JD Hunter, Matplotlib: A 2d graphics environment. *Comput. Sci. & Eng.* **9**, 90–95 (2007).
16. G Klesse, S Rao, MS Sansom, SJ Tucker, Chap: a versatile tool for the structural and functional annotation of ion channel pores. *J. molecular biology* **431**, 3353–3365 (2019).
